# Sustained delivery of NT-3 and curcumin augments microenvironment modulation effects of decellularized spinal cord matrix hydrogel for spinal cord injury repair

**DOI:** 10.1093/rb/rbae039

**Published:** 2024-04-10

**Authors:** Jiaxin Chen, Xing Cheng, Zhengran Yu, Rongli Deng, Rui Cui, Jing Zhou, Houqing Long, Yong Hu, Daping Quan, Ying Bai

**Affiliations:** Guangdong Engineering Technology Research Centre for Functional Biomaterials, Key Laboratory for Polymeric Composite & Functional Materials of Ministry of Education, School of Materials Science and Engineering, Sun Yat-sen University, Guangzhou 510006, China; Department of Spine Surgery, The First Affiliated Hospital of Sun Yat-sen University, Guangzhou 510080, China; Department of Spine Surgery, The First Affiliated Hospital of Sun Yat-sen University, Guangzhou 510080, China; Guangdong Engineering Technology Research Centre for Functional Biomaterials, Key Laboratory for Polymeric Composite & Functional Materials of Ministry of Education, School of Materials Science and Engineering, Sun Yat-sen University, Guangzhou 510006, China; Guangdong Engineering Technology Research Centre for Functional Biomaterials, Key Laboratory for Polymeric Composite & Functional Materials of Ministry of Education, School of Materials Science and Engineering, Sun Yat-sen University, Guangzhou 510006, China; Guangdong Engineering Technology Research Centre for Functional Biomaterials, Key Laboratory for Polymeric Composite & Functional Materials of Ministry of Education, School of Materials Science and Engineering, Sun Yat-sen University, Guangzhou 510006, China; Department of Spine Surgery, The First Affiliated Hospital of Sun Yat-sen University, Guangzhou 510080, China; Department of Orthopedics and Traumatology, Li Ka Shing Faculty of Medicine, The University of Hong Kong, Hong Kong 999077, China; Guangdong Engineering Technology Research Centre for Functional Biomaterials, Key Laboratory for Polymeric Composite & Functional Materials of Ministry of Education, School of Materials Science and Engineering, Sun Yat-sen University, Guangzhou 510006, China; Guangdong Engineering Technology Research Centre for Functional Biomaterials, Key Laboratory for Polymeric Composite & Functional Materials of Ministry of Education, School of Materials Science and Engineering, Sun Yat-sen University, Guangzhou 510006, China

**Keywords:** decellularized extracellular matrix, hydrogel, spinal cord injury, curcumin, neurotrophin-3

## Abstract

Decellularized extracellular matrix hydrogel, especially that derived from spinal cord (DSCM hydrogel), has been actively considered as a functional biomaterial for remodeling the extracellular matrix of the native tissue, due to its unique characteristics in constructing pro-regenerative microenvironment for neural stem cells (NSCs). Furthermore, DSCM hydrogel can provide multiple binding domains to growth factors and drugs. Therefore, both exogenous neurotrophic factors and anti-inflammatory drugs are highly desired to be incorporated into DSCM hydrogel, which may synergistically modulate the complex microenvironment at the lesion site after spinal cord injury (SCI). Herein, neurotrophin-3 (NT-3) and curcumin (Cur) were integrated into DSCM hydrogel for SCI therapy. Due to different affinities to the DSCM hydrogel, NT-3 underwent a controlled release manner, while curcumin released explosively within the first 24 h, followed by rather sustained but slower release. The integration of both NT-3 and curcumin significantly enhanced NSCs proliferation and their neuronal differentiation. Meanwhile, the release of curcumin promoted macrophages polarization into anti-inflammatory subtypes, which further facilitated NSCs differentiation into neurons. The *in situ* injected DSCM + NT3 + Cur hydrogel exerted superior capability in alleviating the inflammatory responses in rat contused spinal cord. Compared to DSCM hydrogel alone, DSCM + NT3 + Cur hydrogel more significantly promoted the recruitment of NSCs and their neuronal differentiation at the lesion site. These outcomes favored functional recovery, as evidenced by the improved hind limb movement. Overall, the bioactive DSCM hydrogel can serve as a multifunctional carrier for cooperatively release of growth factors and drugs, which significantly benefits microenvironment regulation and nerve regeneration after SCI.

## Graphical Abstract

**Figure:**
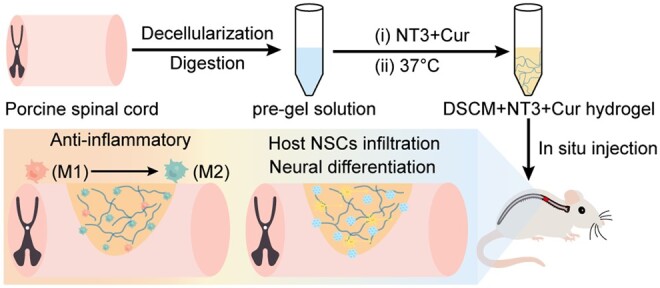

## Introduction

Spinal cord injury (SCI) is a destructive disorder of the central nervous system that causes permanent motor, sensory and autonomic dysfunctions. After acute SCI, a cascade of secondary injuries, such as ischemia, oxidative stress, inflammation and apoptosis, severely hinders nerve regeneration and locomotor functional recovery [1]. Numerous therapeutic strategies, including medication, physical stimulation, cell therapy, gene therapy and biomaterial transplantation, have been developed which attempted to improve the devastated microenvironment, stimulate nerve regeneration and rebuild neural circuits at the injury sites [2, 3]. However, the outcomes of such therapies are still far from satisfaction regarding restoration of both intraneuronal and regenerative microenvironment balances [4]. Therefore, refinement the stem cell niches through regulating the inflammatory responses in the acute phase, as well as the recruitment and directed differentiation of endogenous neural stem cells (NSCs), has both become key to breaking through the bottleneck of SCI treatments [5, 6].

As a sort of natural biomaterial, decellularized extracellular matrix (dECM) retains the biophysical structures, components of extracellular matrix (ECM) from the native tissues and tissue-specific bioactive factors [7, 8]. Furthermore, the injectable dECM hydrogels can be easily prepared by following the consecutive processes of enzymatic digestion, pH neutralization and ionic balance [9]. Consequentially, the dECM hydrogels derived from parts of nervous systems, such as the spinal cord [10], peripheral nerve [11], optic nerve [12] and brain [13], have been transplanted and investigated for SCI repair. Previously we reported that the dECM hydrogel derived from the spinal cord (DSCM hydrogel) exhibited unique tissue-specific advantages in potentiating neural stem/progenitor cell (NSPC) proliferation, migration and especially neuronal differentiation when compared with the decellularized peripheral nerve matrix hydrogel [10]. Moreover, the DSCM hydrogel also contributes to maintaining the immaturity of NSPC-differentiated astrocytes and their pro-regenerative phenotype [14]. However, considering the complex environment at the lesion site after SCI, the injected DSCM hydrogel alone cannot serve to balance the acute inflammatory responses and nerve regeneration precisely and sequentially. Therefore, it is necessary to introduce functional drugs and neurotrophic factors into the DSCM hydrogel for effective regulation of inflammatory responses, meanwhile, facilitating the endogenous NSCs recruitment and differentiation.

Curcumin, a natural compound extracted from *Curcuma longa*, is well acknowledged for its anti-inflammatory and antioxidant pharmacological effects [15, 16]. Previous studies reported that during the acute inflammation after SCI, the introduced curcumin effectively reduced macrophages infiltration into the injury sites, and induced pro-inflammatory (M1) to anti-inflammatory (M2) macrophage transformation, providing a favorable microenvironment for NSCs migration [17, 18]. Additionally, curcumin contributed to the regulation of astrocyte phenotype, inhibited glial scar formation and facilitated NSCs differentiation [19]. However, the effectiveness of curcumin alone has been quite limited, either by injection or oral administration, owing to its hydrophobic nature. A proper carrier is highly desired for *in situ* delivery of curcumin and its sustainable release at the lesion site, which also protects the drug from cerebrospinal fluid circulation clearance. Besides curcumin, introduction of exogenetic neurotrophic factors can further enhance endogenous nerve regeneration and promote functional recovery of the injured spinal cord. Neurotrophin-3 (NT-3) has been recently demonstrated to recruit endogenous NSCs to proliferate, migrate and differentiate into neurons at lesion sites. The administrated NT-3 also inhibits axonal degeneration and forms nascent neural networks for functional recovery [20–23]. Hence, the sustained delivery of NT-3 potentially plays a pivotal role in facilitating nerve regeneration after SCI. Interestingly, it has been noticed that neurotrophic factors often underwent control release when integrated into dECM hydrogels, which was mostly attributed to their specific binding affinity to heparan sulfate proteoglycan, an intrinsic component in the ECM of nerve tissues [24].

Taking advantage of the tissue-specific and injectable DSCM hydrogel, both curcumin (Cur) and NT-3 (NT3) were both integrated and co-delivered to the lesion site, which were expected to modulate the SCI-induced inflammatory microenvironments into pro-regenerative ones toward endogenous regeneration. In this study, a multifunctional DSCM + NT3 + Cur hydrogel was prepared by incorporating curcumin and NT-3 into DSCM pre-gel solution simultaneously, which can gel at 37°C or under physiological condition. Rheological property, morphological features, curcumin/NT-3 release and degradation behavior of the DSCM + NT3 + Cur hydrogel were characterized, which was further compared with the DSCM hydrogel alone, DSCM + NT3 and DSCM + Cur hydrogels. The dosages of NT-3 and curcumin in DSCM hydrogel were examined and optimized by cytocompatibility tests. Furthermore, to assess the corresponding bioactivities of the hydrogels, NSCs proliferation, differentiation and regulation of macrophage phenotypes were evaluated *in vitro*. Finally, the pro-regenerative efficacy of the hydrogel was examined using a spinal cord contusion model in rats, focusing on the inflammatory cells, endogenous NSCs recruitment and their differentiation through histological analysis and immunofluorescence staining. The restoration of motor function was estimated by behavioral assessment. The aforementioned evaluations revealed that the DSCM hydrogel served as a suitable cargo and pro-regenerative biomaterial for sustained co-delivery of drugs and growth factors, which facilitated nerve regeneration and functional recovery after SCI. (A list of abbreviations is attached in Supplementary Table S1.)

## Materials and methods

### Ethics

All experimental protocols and animal handling procedures were approved and supervised by the Animal Care and Use Committee of Sun Yat-sen University (approval No. 2022002238) and were in line with the Laboratory Animal Regulations of Guangdong Province (2010 No. 41). Animal welfare complied with the Laboratory Animal Guideline for Ethical Review Animal Welfare, General Administration of Quality Supervision, Inspection and Quarantine of People’s Republic of China/Standardization Administration of China (GB/T 35892-2018).

### Decellularization

The porcine derived decellularized spinal cord matrix (DSCM) was prepared according to previously reported protocol [10], approved by the Animal Ethics Committee of the First Affiliated Hospital of Sun Yat-sen University. Briefly, the dissected fresh spinal cord of male landrace was first sterilized with 2% penicillin/streptomycin for 6 h, then decellularized by 3% v/v Triton X-100 for 1 h and 4% v/v sodium deoxycholate for another hour at 4°C. After decellularization, the spinal cord was rinsed by sterile water for three times, followed by freeze-drying, and then, pulverized into powder, stored at −40°C until use.

### Preparation of DSCM + NT3, DSCM + Cur and DSCM + NT3 + Cur hydrogels

The DSCM powder was digested in an acidic solution (0.01 M HCl) containing pepsin (1 mg/ml) for 6 h at 25°C. The residual particulates were removed by centrifugation at 30 000 rpm for 1 h at 4°C. The pH of the digest solution was gently adjusted to 8.0 using 1.0 M NaOH for terminating digestion, followed by addition of 0.01 M HCl until the pH value reached ∼7.4. NT-3 (PeproTech, USA) and curcumin (Admas-beta, China) were either separately or both mixed into the above DSCM pre-gel solution, then isotonically balanced using 10 × PBS solution. The pre-gel solution was gelled at 37°C to obtain DSCM + NT3, DSCM + Cur and DSCM + NT3 + Cur hydrogels, respectively.

### Rheological characterization

The rheological properties of the hydrogels were assessed by a rheometer (Kinexus Pro + Rheometer, Malvern Instruments Ltd, UK) equipped with a parallel plate to obtain the sol-gel transition temperature and moduli. All samples (0.25 ml) were applied to the lower plate of the rheometer. Temperature sweeps were performed to collect storage (*G*′) and loss (*G*″) moduli at a constant frequency of 1 Hz, and oscillation temperature increased from 20 to 45°C at rate ∼2.5°C/min.

### Scanning electron microscopy

To observe the morphologies of the prepared DSCM hydrogels, the samples were pre-fixed in 2.5% glutaraldehyde at 4°C for 6 hr, followed by rinsing in deionized water for three times, and dehydrated in a graded series of alcohol (20%, 40%, 60%, 80% and 100% ethanol) for 1 h per rinse. Then, the samples were immersed in deionized water for three times (water changed every 30 min) and subjected to lyophilization. The lyophilized samples were torn to expose the fractured surface and pasted on a conductive tape. After coating with platinum, the samples were characterized by scanning electron microscopy (SEM, S-4800, HITACHI, Japan) at 10 kV voltage.

### Release profiles of NT-3 and curcumin in DSCM hydrogel

The standard curve line of curcumin was constructed by recording the absorbance of serial dilutions of curcumin solution of known concentration (0.05, 0.1, 0.2, 0.4, 0.6, 0.8, 1, 2, 4, 6, 8, 10 μg/ml) containing 2.5% (w/v) Tween-80 PBS at 429 nm. Curcumin-loaded DSCM hydrogel (DSCM + Cur) was prepared as described above and used for the characterization of curcumin release profile. One milliliter DSCM + Cur hydrogel was placed into 6 ml PBS solution containing 2.5% (w/v) Tween-80 at 37°C. At the predetermined time points, an aliquot of the released mixture (1 ml) was withdrawn and replaced with an equal volume of fresh media. The concentrations of released curcumin were examined by the UV–Vis spectrophotometer (Lambda 950, Perkin Elmer, UK). Each test was carried out for five times, and average values were plotted.

To determine the NT-3 release profile, 200 μl DSCM hydrogel containing 300 ng/ml NT-3 (DSCM + NT3 hydrogel) was placed into 500 ml PBS at 37°C. The supernatant was collected at the predetermined time points, which was replaced by an equal volume of fresh PBS, and then, stored at −20°C. The concentration of NT-3 was determined according to the instructions of the ELISA kit (Thermofisher, USA). Each experiment was carried out for five times, and the cumulative release rate of NT-3 was calculated to obtain the release profile.

### Degradation

To assess the stability under 37°C, the degradation behaviors of the prepared hydrogels were evaluated by immersing in two different media, respectively, one was PBS solution alone, and the other was PBS solution containing 2 U/ml collagenase type I. At each predetermined time points, the residual hydrogels were weighted after obliterating the surface water. The percentage of residual mass was calculated using the following Eq. (1),

(1) $$\mathrm{Residual}\;\mathrm{mass}\;\left(\%\right)=\frac{W_{t}^{'}}{W_{0}^{'\;}}\;\times \;100\%$$

where *W_0_′* represents the initial weight of the hydrogels, and *W_t_′* represents the weight at each time point.

### Isolation and culture of neural stem cells (NSCs)

The Laboratory Animal Center of Sun Yat-sen University supplied all the Sprague–Dawley (SD) rats used in this study. In brief, the hippocampus was extracted from an E14.5 embryo SD rat and placed in an ice-cold medium. After separating the endocranium and blood vessels, the tissues were dissociated gently into cell clumps and then centrifugated at 1000 rpm for 5 min. The cell suspension was cultured in serum-free DMEM/F12 medium (Gibco, USA) containing 2% B27 (Invitrogen, USA), 20 ng/ml EGF (Peprotech Asia, Israel), 20 ng/ml FGF2 (Peprotech Asia, Israel) and 1% penicillin-streptomycin (Invitrogen, USA). After 3–4 days of culture, the resulting NSC spheres were centrifuged, digested into single cells using Accutase (Gibco, USA) and suspended in the fresh proliferation medium for the following cell culture experiments. All the NSCs used in this study were from the third passage.

### Cytocompatibility

DSCM hydrogels containing curcumin (30, 125, 500 ng/ml) and NT-3 (100, 300, 500 ng/ml) were prepared, respectively. The NSCs were cultured to the third generation and transferred to the hydrogels at 1 × 10^5^ cells per well, then cultured in a proliferation medium (DMEM/F12, 2% B27, 1% GlutaMax). The viability of NSCs was examined using Live/Dead assay (KeyGEN BioTECH, Nanjing, China) after 3 days of culture. The live and dead cells were imaged using an inverted fluorescence microscope (Axio Observer Z1, Carl Zeiss, Germany). The images were analyzed using ImageJ software (NIH, USA). The viability of NSCs on each hydrogel was calculated and represented using the proportion of live cells to the total cells. The proliferation of NSCs was detected by cell counting kit-8 (CCK-8, Dojindo, Japan). The cells were incubated in the medium containing 10% CCK-8 for 2 h in the 37°C humidified incubator. The absorbance of the stained solution was measured at 450 nm using a microplate reader (Synergy H1, Biotek, VT, USA).

To evaluate the hydrogel-mediated hemolysis, sodium citrate rat whole blood was centrifuged at 1000 rpm for 10 min, then saline-rinsed for three times and diluted to the concentration ∼5% (v/v). 0.5 ml of each hydrogel and 1.0 ml blood (5% v/v) were mixed in a 2 ml tube, followed by incubation at 37°C for 4 h. Then, the blood was transferred into a 1.5 ml tube and centrifuged at 3000 rpm for 10 min. The absorbance of the supernatant was measured at wavelength ∼540 nm. Deionized water represented positive control and saline represented negative control. Hemolytic quantification was calculated using the following Eq. (2),

(2) $$\mathrm{Hemolysis}\;\left(\%\right)=\frac{\left(A_{p}-A_{b}\right)}{\left(A_{t}-A_{b}\right)}\times 100\%$$

where *A_p_* represents the absorbance of each hydrogel, *A_t_* represents the absorbance of positive control and *A_b_* represents the absorbance of negative control.

### *In vitro* NSCs proliferation and differentiation

To assess the proliferation of NSCs, the NSC spheroids were, respectively, seeded on the DSCM, DSCM + NT3, DSCM + Cur and DSCM + NT3 + Cur hydrogels and cultured in proliferation medium (DMEM/F12, 2% B27, 1% GlutaMax). The NSCs were cultured for 3 days, and the proliferating cells were labeled using the commercial EdU assay Kit (RiboBio, China, C10310-3). To evaluate the expressions of NSCs markers, the NSCs were fixed in 4% paraformaldehyde for 20 min, then, subjected to immunofluorescence staining.

To evaluate the differentiation of NSCs, the isolated NSCs were cultured on DSCM, DSCM + NT3, DSCM + Cur and DSCM + NT3 + Cur hydrogels at a density of 1 × 10^5^ cells per well, respectively, and cultured in NSCs differentiation medium (Neurobasal medium, 2% B27, 1% GlutaMax) at 37°C in a humidified incubator with 5% CO_2_. The media were replaced every other day. After culture for 14 days, the NSCs were fixed using 4% paraformaldehyde for 20 min, and then, subjected to immunofluorescence staining.

### Identification of macrophage phenotypes and NSCs differentiation

Murine RAW 264.7 macrophages (Procell Life Science & Technology Co., Ltd, China, CL-0190) were cultured in DMEM medium with 10% fetal bovine serum (Procell Life Science & Technology Co., Ltd, China, CM-0190). RAW 264.7 cells were seeded on 6-well plates at 1 × 10^5^ cells/well and cultured for 24 h at 37°C in a humidified incubator with 5% CO_2_. Then, the cells were treated with DMEM medium containing 100 ng/ml LPS (lipopolysaccharide, sigma, USA) and 20 ng/ml IFN-γ (Interferon gamma, sigma, USA) for 24 hr. After the stimulation, the macrophages (at a concentration of 5 × 10^4^/ml) were digested and seeded on the hydrogels. After 3 days of culture, cells were fixed using 4% paraformaldehyde for 20 min, and subjected to immunofluorescence staining.

To further quantify the secretion of pro-inflammatory and anti-inflammatory cytokines, RAW 264.7 cells (density ∼ 5 × 10^4^/ml) cultured on the hydrogels were pre-treated with DMEM medium containing 100 ng/ml LPS and 20 ng/ml IFN-γ for 24 hr, respectively. After 24-h stimulation, the supernatant of each culture medium was collected and centrifuged at 1000 × g for 10 min to remove cellular debris. Secreted cytokines were examined by using Mouse TNF-α (Elabscience, China), Mouse IL-6 (Elabscience, China), Mouse IL-10 (Elabscience, China) and Mouse TGF-β (Elabscience, China) Cytokine ELISA kit, respectively, according to the manufacturer’s protocols. The stimulated RAW 264.7 cells cultured on hydrogel-free tissue culture plates were used as the control group (denoted as the LPS group).

For evaluation of NSCs differentiation in inflammatory environment, the isolated NSCs (1 × 10^5^ cells/well) were cultured on different hydrogels using a mixed medium which was prepared by the supernatant of LPS and IFN-γ stimulated macrophages mixing with neurobasal medium at 1:1 ratio. After 14 days, the cultured NSCs were fixed with 4% paraformaldehyde for 20 min for immunofluorescence staining.

### Immunofluorescence staining

The NSCs were permeabilized and blocked with 0.5% Triton X-100 and 5% donkey serum for 30 min at room temperature, then incubated with primary antibodies (Supplementary Table S2) overnight at 4°C. After 12-h incubation, the cells were rinsed with PBS solution and incubated with secondary antibodies (Supplementary Table S2) for 1 h at 37°C. The immunostained cells were rinsed with PBS for three times, prior to the nuclei staining using DAPI (5 μg/ml). The labeled cells were observed using a confocal microscope (Zeiss LSM 880, Germany). The protein expressions were analyzed using ImageJ (NIH, USA) and GraphPad Prism 8 (GraphPad Software, USA).

### Spinal cord contusion model

Adult female SD rats (220–250 g) were purchased from the Laboratory Animal Center of Sun Yat-Sen University. All animals were housed in an SPF animal facility and fed for a week, and then, the rats were randomly divided into three groups for *in vivo* study: SCI group (*n* = 15), DSCM group (*n* = 15), DSCM + Cur + NT3 group (*n* = 15). Before surgery, each rat was anesthetized by intraperitoneal injection of 1% sodium pentobarbital (30 mg/kg body weight) and laminectomy was performed aseptically at T9 - T10 to expose the spinal cord. The spinous processes at T8 and T11 were fixed with clamps to prevent movement during contusion. During the period of anesthesia, body temperature was kept constant using a temperature-controlled heating pad. The spinal cord contusion rat model was constructed using a spinal cord impactor (Impactor Model II, NYU, USA). The T9 contusion injury was produced by dropping a 10-g rod with a tip diameter of 2.5 mm from 25 mm height. After the contusion, the spine was edema, the tail swung spasmodically and both lower limbs showed retraction flapping, exhibiting delayed paralysis. After hemostasis, 10 μl DSCM + Cur + NT3 hydrogel (pH 7.4, for the DSCM + Cur + NT3 group), DSCM hydrogel alone (pH 7.4, for the DSCM group) and 0.9% saline (for the SCI group) were gently administrated into the lesion sites, respectively, using a 10 μl microinjector (tip diameter: 0.5 mm, tip length: 55 mm, Shanghai Gaoge Industry and Trading Co. LTD, Shanghai, China). Then, the muscles and skin were sutured consecutively in corresponding layers. After the surgery, the rats were intramuscularly injected with penicillin (50 000 U/kg per day) for 1 week and manual micturition twice a day until the restoration of bladder function.

### Locomotor function recovery

For the evaluation of locomotor functions of paralyzed hindlimbs after contused SCI, open-field locomotion was evaluated using the BBB locomotor rating scale [25]. The hindlimb movement was scored from 0 (= total paralysis) to 21 (= normal locomotion), and the test was performed weekly from the first to eighth week after injury. All locomotor assessments were performed by two observers blinded to the group allocation. The weekly scores were statistically analyzed by GraphPad Prism 8 (GraphPad Software, USA).

The inclined-grid climbing test was employed to assess the accuracy of foot coordination during locomotion [26]. When the body of rats was held at two different positions on the board for 5 s, the maximum angle between the board and the ground was the angle to be recorded. The inclined-grid climbing test was carried out by two observers blinded to the group allocation 8 weeks after SCI.

### Tissue preparation, histological analysis and immunofluorescence staining

Two- and eight-weeks post-surgery, the rats in all experimental groups were deeply anesthetized with sodium pentobarbital (i.p., 30 mg/kg) and transcardially perfused with saline (0.9%, 500 ml) and 4% paraformaldehyde (PFA, 500 ml), consecutively. The thoracic segments of T9–T11 were dissected and post-fixed in 4% paraformaldehyde overnight at 4°C. The dissected tissues were dehydrated with 20% v/v sucrose for 24 hr, followed by 30% sucrose for another 48 h at 4°C and embedded in optimal cutting temperature compound (OCT, SAKURA, Japan). All samples were longitudinally cut into 10-μm thick cryosections with a freezing microtome (Leica CM1950, Germany). For histological staining, the cryosections were subjected to hematoxylin and eosin (H&E) and Masson trichrome staining, respectively. Images were acquired using AxioScan Z1 (Zeiss, Germany). The cavity area at the lesion sites was estimated by ImageJ software (NIH, USA).

To assess the biosafety of the DSCM and DSCM + NT3 + Cur hydrogels *in vivo*, the spleens, kidneys and livers of both hydrogel-injected groups were dissected, washed with normal saline and fixed in 4% paraformaldehyde solution, then subject to OCT embedding and pathological sectioning. H&E staining was used for histological analysis of these vital organs with comparison against those dissected from the healthy rats.

For immunostaining, the slides were permeabilized with 0.5% Triton X-100, blocked with 5% donkey serum for 1 h at room temperature, and then, incubated with primary antibodies (Supplementary Table S2) overnight at 4°C. After rinsing with PBS solution, the sections were incubated with secondary antibodies (Supplementary Table S2) for 1 h. Then, the sections were washed with PBS solution for three times, nuclei were stained with DAPI (5 μg/ml) for 20 min at room temperature and covered with glass slips with the anti-fade fluorescence mounting medium. The sections were captured by AxioScan Z1 (Zeiss, Germany) in fluorescence mode.

Five randomly selected fields were captured to assess the inflammatory cell infiltration at the injury sites 2 weeks after surgery, and the number of CD206+/CD68+ cells and iNOS+ cells were counted. Similarly, to determine the migration of endogenous NSCs, the number of SOX2+ cells was counted. In addition, the fluorescence intensity of Tuj1+, GFAP+, MAP2+ and NF200+ signals were analyzed using ImageJ, respectively.

### Statistical analysis

All statistical analyses were performed using the statistical software GraphPad Prism 8 (GraphPad Software, USA). Data were shown as means ± standard deviation. The data were analyzed using one-way analysis of variance (One-Way ANOVA) or two-way repeated measures of variance (Two-Way ANOVA). The values were considered significantly different at *P *<* *0.05.

## Results and discussion

### Physical properties of the hydrogels and sustained NT-3/curcumin release

**Figure 1.**
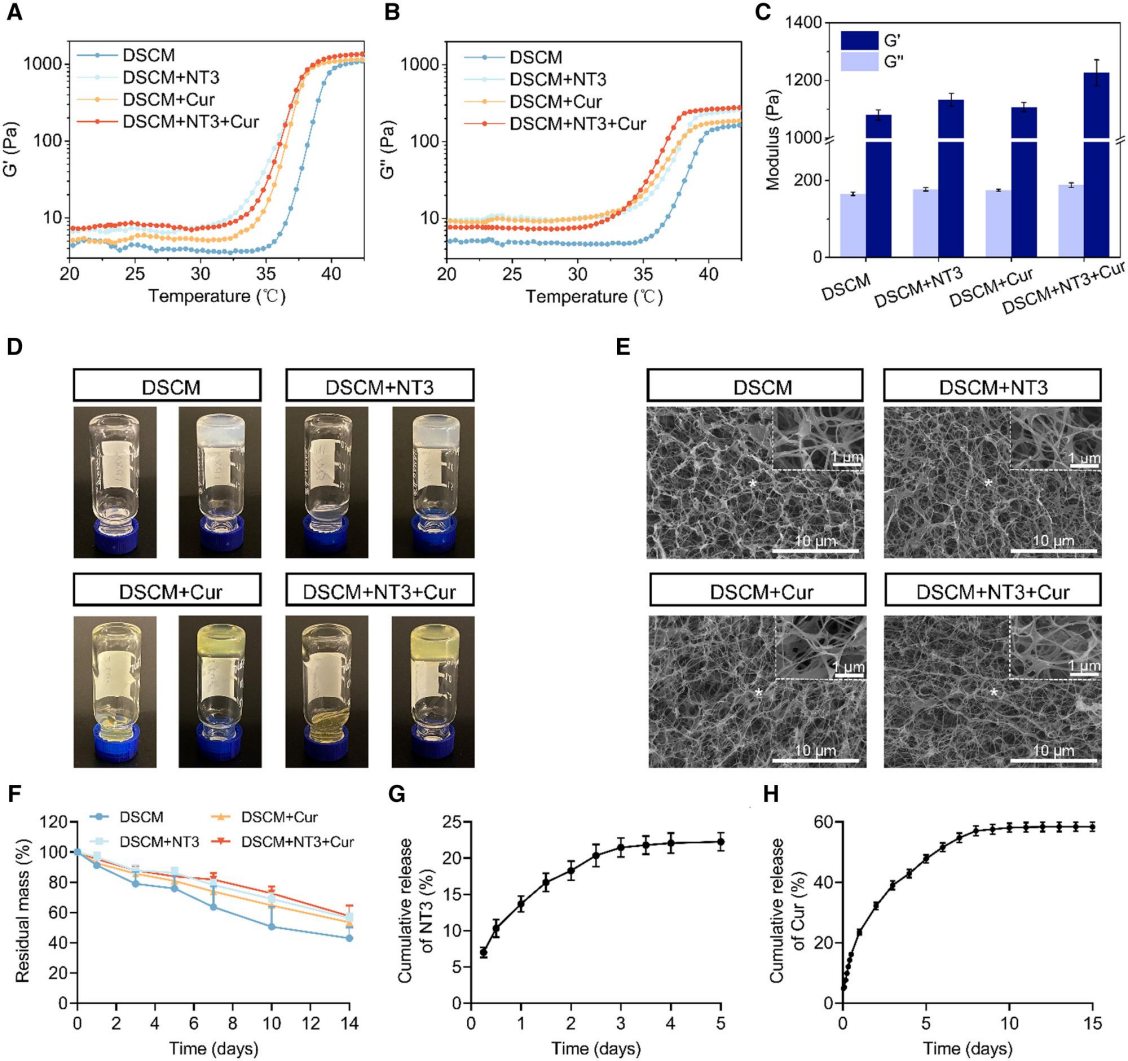
Characterizations of the DSCM, DSCM + NT3, DSCM + Cur and DSCM + NT3 + Cur hydrogels. (**A**) Storage moduli (*G′*) and (**B**) loss moduli (*G*″) of the hydrogels measured by rheometer at temperature ranging from 20°C to 45°C. (**C**) Statistical analysis of both *G′*s and *G*″s of the hydrogels. (**D**) The pre-gel solutions (left-hand side) in inverted vials, and their corresponding hydrogels (right-hand side) formed after sol-gel transition at 37°C, respectively. (**E**) SEM micrographs of the ECM-like nanofibrous structures in the DSCM, DSCM + NT3, DSCM + Cur and DSCM + NT3 + Cur hydrogels, scale bars = 10 μm. The insets in the upper right corners show the zoom-in images of the corresponding areas marked by asterisks, scale bars = 1 μm. (**F**) Degradation behaviors of the hydrogels immersing in collagenase type I containing PBS solution at 37°C for 2 weeks. (**G**) Cumulative NT-3 release from DSCM hydrogel, assessed by ELISA. (**H**) Cumulative curcumin release from DSCM hydrogel, tested using UV–Vis spectrophotometer.

After decellularization, the DSCM powder underwent digestion, pH neutralization and ionic balancing to obtain the DSCM pre-gel solution. When the temperature was lower than 35°C, the DSCM pre-gel solution was a viscous fluid with very low storage modulus (*G′*) and loss modulus (*G″*). Once the temperature exceeded 35°C, both *G′* and *G″* of DSCM-gel increased significantly, which underwent sol-gel transition (Figure 1A and B). Interestingly, the addition of NT-3 and curcumin slightly shortened the gelation time and increased both moduli compared to the DSCM-gel alone (Figure 1C). As a result, it was easily noted that the liquid-like pre-gel solution (the ones on the left-hand side in Figure 1D) of each sample turned into solid-like hydrogels (the ones on the right-hand side in Figure 1D). The DSCM + Cur and DSCM + NT3 + Cur hydrogels exhibited yellow-ish color owing to the intrinsic fluorescence of curcumin [27]. Microscopically, similar ECM-like nanofibrous ultrastructure were evident through SEM characterization on all the hydrogel specimens (Figure 1E). NT-3 was uniformly dispersed in DSCM pre-gel solution by vortex mixing before gelation. For the curcumin, no obvious precipitation was identified inside the Cur-loaded hydrogels. The diameter of the nanofibers showed no significant difference between DSCM hydrogel and DSCM + NT3 hydrogel, while the incorporation of curcumin showed slight reduction in fiber diameter (Supplementary Figure S1). The above characterizations of physical and morphological properties indicated that the NT-3 and/or curcumin containing DSCM hydrogels retained the properties of the original dECM hydrogel. To assess the effects of integrated NT-3 and curcumin on hydrogel degradation performance, all the specimens were immersed in PBS solution with and without collagenase type I, respectively. When immersed in pristine PBS solution, all the hydrogels degraded slowly during the first 7 days, then, slightly faster thereafter. Overall, the degradation profiles of all the hydrogels were close to each other, and more than 70% of the original mass retained after 2 weeks (Supplementary Figure S2). To better mimic the *in vivo* environment, collagenase type I was supplemented to PBS solution, resulting in much faster degradation of all hydrogels, especially for DSCM hydrogel alone (Figure 1F). Comparatively, the NT-3 and/or curcumin containing hydrogels exhibited much slower degradation behavior. The addition of NT-3 and curcumin apparently improved the stability of the DSCM hydrogel.

Furthermore, the release profiles of DSCM-gel were examined at predetermined time points, with and without NT-3 and curcumin integration. It was noted that the neurotrophic factor NT-3 exhibited a slow but sustained release from DSCM hydrogel (Figure 1G). However, curcumin showed a burst release within the first day, then followed a more controlled release profile until reached a plateau, ie an equilibrium state, after ∼10 days (Figure 1H). DSCM hydrogel retains many functional ECM components from the native tissue, such as type I collagen and proteoglycans, offering multiple binding sites to bioactive factors and drugs. The controlled release of NT-3 was attributed to the strong binding affinity between growth factor and specific ECM components in DSCM hydrogel, such as heparan sulfate proteoglycan [28–33]. Curcumin was released much faster (burst release) at the beginning, mostly owing to its hydrophobic nature that might be repelled from the hydrogel. But as a natural derived polyphenol, curcumin also contains abundant phenolic hydroxyl groups that potentially attach to the collagen and elastin contents in the dECM through the formation of hydrogen bonds [34, 35]. The encapsulated curcumin can further serve as a crosslinker in the DSCM hydrogel, not only promotes the stability of the collagen matrix, but also imparts therapeutic properties to the hydrogel [36, 37]. The difference between NT-3 and curcumin release behaviors was expected to match with the pathologic process during SCI repair, in which the anti-inflammatory curcumin is more required during the acute phase of inflammation, while NT-3 continuously contributes to nerve regeneration at the later stages.

### Biocompatibility of the hydrogels and NT-3/curcumin dosage selection

**Figure 2.**
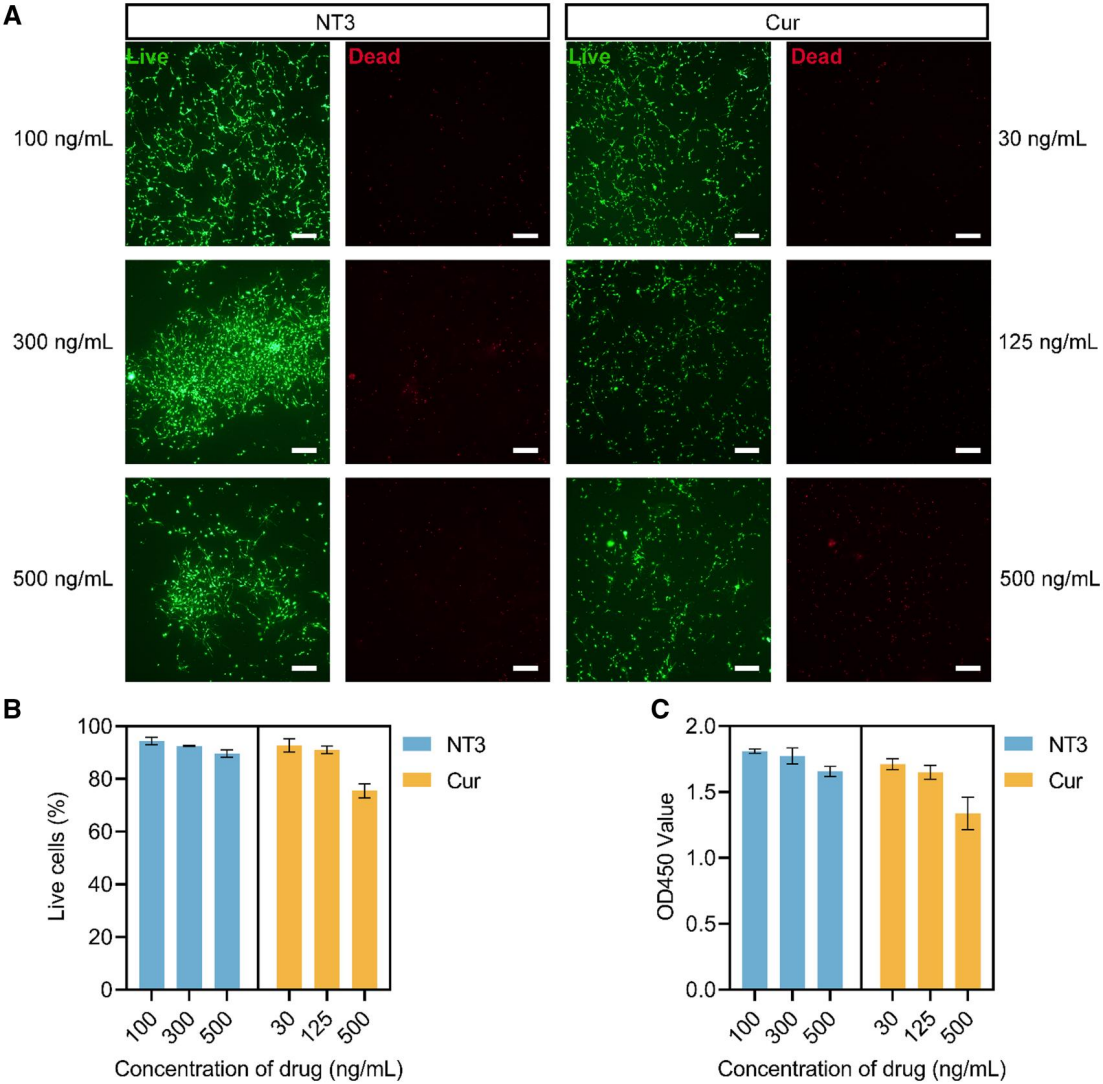
Viability and proliferation of the NSCs cultured on the DSCM hydrogels containing different dosages of NT-3 and curcumin, respectively. (**A**) Live/dead staining of the NSCs cultured on DSCM + NT3 and DSCM + Cur hydrogels for 3 days. Scale bars = 200 μm. (**B**) Quantification of NSCs viability on the DSCM + NT3 and DSCM + Cur hydrogels, assessed by live/dead assay. (**C**) OD values of the NSCs cultured on the DSCM + NT3 and DSCM + Cur hydrogels after 3 days, assessed by CCK-8 assay. Data were presented as the mean ± SD. *n* = 3 for all groups.

To verify the cytocompatibility of the DSCM + NT3 and DSCM + Cur hydrogels with different dosages, primary NSCs were seeded and cultured on the DSCM hydrogels with predetermined NT-3 (100, 300 and 500 ng/ml) and curcumin (30, 125 and 500 ng/ml) concentrations, respectively. The cultured NSC spheroids were identified by phalloidin-staining, as well as immunofluorescence staining using biomarkers SOX2 and Nestin (Supplementary Figure S3). The viability and proliferation of NSCs were evaluated by Live/Dead and CCK-8 assays after 3 days of culture. It was evident that the NSCs accommodated well on the DSCM + NT3 hydrogels with all three different NT-3 concentrations, which exhibited high cell viability (>90%). Notably, the number of NSCs slightly decreased when the concentration of NT-3 was 500 ng/ml (Figure 2A). Since the burst release of curcumin was realized during the first 24 h, the viability of the NSCs was lower (<80%) when the curcumin concentration was 500 ng/ml in the DSCM + Cur hydrogel, compared with those smaller dosages (Figure 2B). Results by CCK-8 assays implicated that all the NT-3 containing hydrogels showed high bioactivity in facilitating NSCs proliferation (Figure 2C). Meanwhile, the NSCs on the DSCM + Cur hydrogel with higher curcumin dosage resulted in lower OD value, hence, the concentration of curcumin was required to be optimized. Considering both the cytocompatibility and dose efficacy, the concentrations of NT-3 and curcumin were determined to be 300 ng/ml and 125 ng/ml, respectively, in the DSCM + NT3 and DSCM + Cur hydrogels, as well as in the DSCM + NT3 + Cur hydrogel, for the following *in vitro* and *in vivo* experiments.

**Figure 3.**
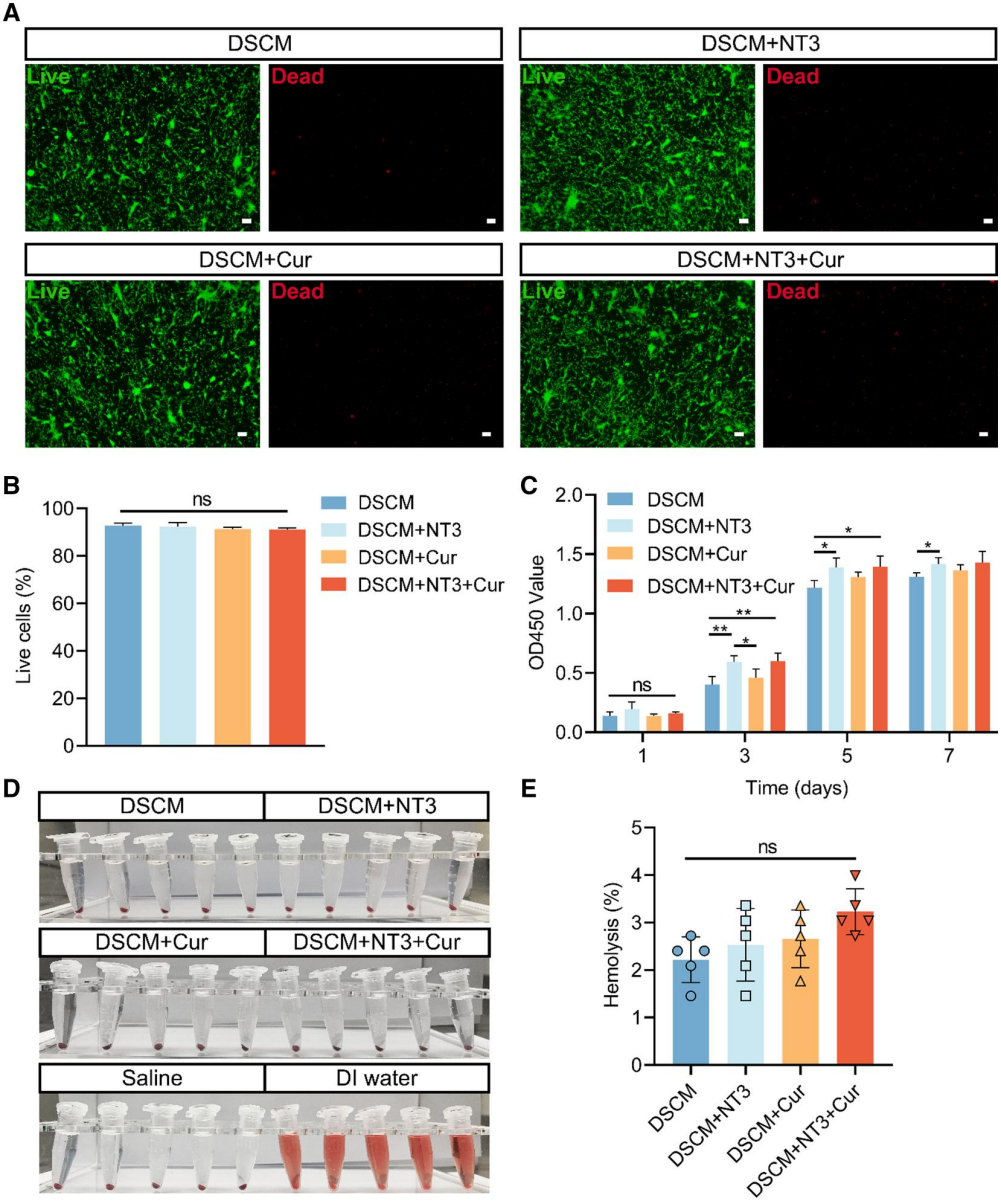
Biocompatibility characterizations of the drug-loaded hydrogels. (**A**) Live/dead staining of the NSCs cultured on DSCM, DSCM + NT3, DSCM + Cur and DSCM + NT3 + Cur hydrogels for 7 days. Scale bars = 100 μm. (**B**) Quantification of NSCs viability after 7 days of culture and live/dead assay. (**C**) OD values of the NSCs cultured on the DSCM, DSCM + NT3, DSCM + Cur and DSCM + NT3 + Cur hydrogels after 1, 3, 5 and 7 days of culture, tested by CCK-8 assay. (**D**) Hemolytic characterization and (**E**) quantification of the hemolysis rate of the hydrogels. Data were presented as the mean ± SD. *n* = 5 for all groups. The statistical significance was calculated by one-way ANOVA with a Tukey’s multiple comparison for analyzing live cell (%) and hemolysis (%) or two-way ANOVA with a Tukey’s multiple comparison for analyzing OD450. ns not significant, *P <* *0.05, **P <* *0.01.

To further evaluate the biocompatibility of each drug-loaded hydrogels, NSCs were cultured on DSCM, DSCM + NT3, DSCM + Cur and DSCM + NT3 + Cur hydrogels for a longer period, respectively. Both results from Live/Dead staining (Figure 3A and B) and CCK-8 assay (Figure 3C) indicated that all hydrogels exhibited good cytocompatibility and capable of facilitating NSCs proliferation after 7 days of culture. Further hemolytic analysis revealed that all hydrogels exhibited very low rate of hemolysis (below 4%, Figure 3D and E), which may highly reduce the risk of hemolytic reactions during *in vivo* administration.

### DSCM + NT3 + Cur hydrogel promotes NSCs proliferation and neuronal differentiation

Previously, it was reported that DSCM hydrogel alone contributes actively in facilitating NSCs proliferation and differentiation into neurons [10]. Considering the functions of neurotrophic factors, the integrated NT-3 in the DSCM hydrogel was expected to be even more beneficial for the survival and proliferation of NSCs. To verify this effect, primary NSCs were cultured on the DSCM, DSCM + NT3, DSCM + Cur and DSCM + NT3 + Cur hydrogels for 3 days, and immunofluorescence staining was performed, biomarker EdU was used for labeling the proliferating cells (Figure 4A). It was noted that a relatively larger amount of Nestin+ NSCs were co-stained by EdU on both DSCM + NT3 and DSCM + NT3 + Cur hydrogels, compared to those observed on the DSCM hydrogel. Statistical analysis further confirmed that the NT-3 containing hydrogels facilitated NSCs proliferation with or without the presence of curcumin (Figure 4B). The results indicated that, despite the highly bioactive DSCM hydrogel, the sustained release of NT-3 still exhibited its advantages in promoting NSCs proliferation, and such benefit was not influenced by the co-integrated curcumin.

**Figrue 4.**
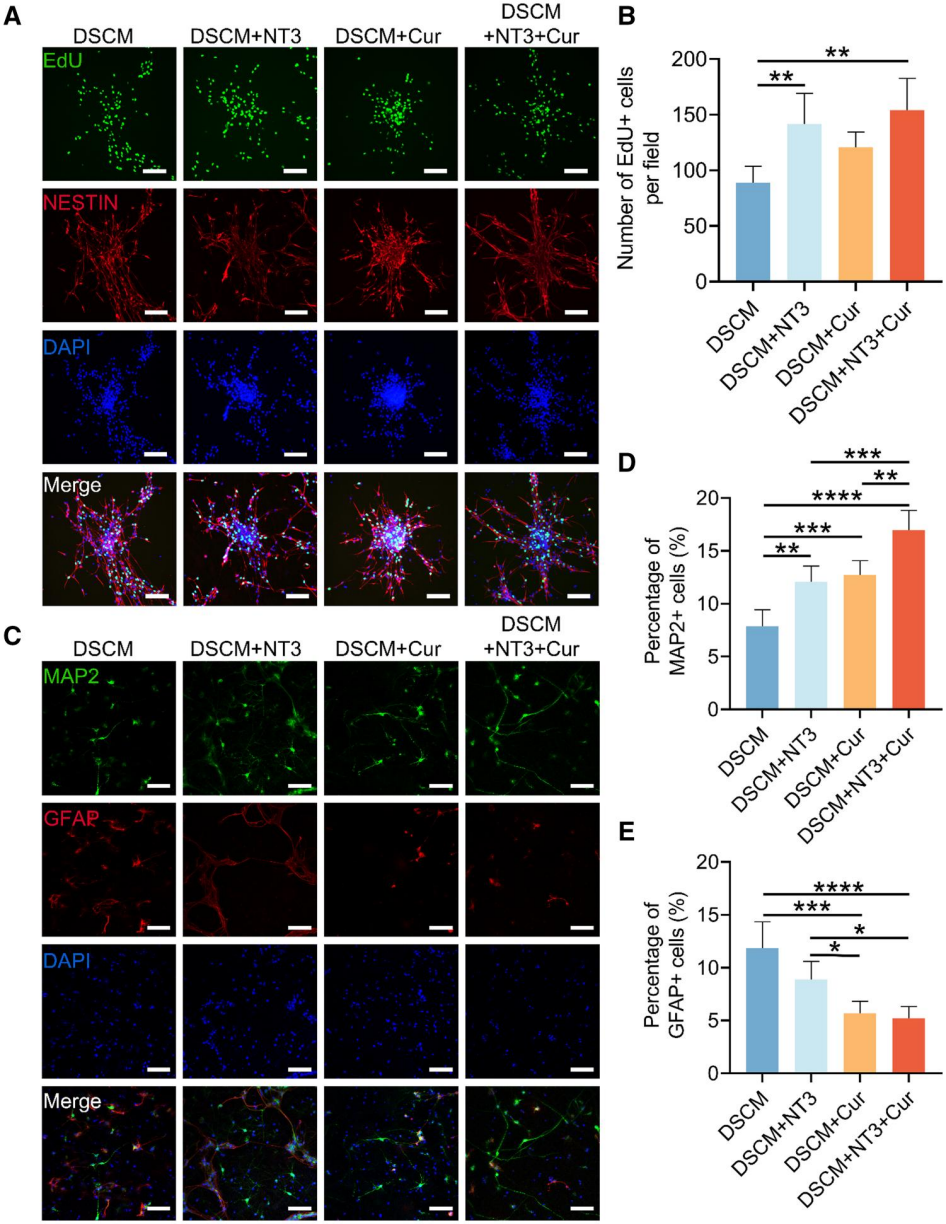
Proliferation and differentiation of the NSCs cultured on the DSCM, DSCM + NT3, DSCM + Cur and DSCM + NT3 + Cur hydrogels, respectively. (**A**) Immunofluorescence staining of the NSCs cultured on the hydrogels for 3 days, using biomarkers EdU, Nestin and DAPI, scale bars = 100 μm. (**B**) Number of EdU+ cells per visual field after 3 days of culture (*n* = 5). (**C**) Immunofluorescence staining of the NSCs cultured on the hydrogels for 14 days, using biomarkers MAP2, GFAP and DAPI, scale bars = 100 μm. (**D**) Percentage of MAP2+ cells among all the cells cultured on the hydrogels (*n* = 5). (**E**) Percentage of GFAP+ cells among all the cells cultured on the hydrogels (*n* = 5). Data were presented as the mean ± SD. The statistical significance was calculated by one-way ANOVA with a Tukey’s multiple comparison. *n* = 5 for all groups; *P <* *0.05, **P <* *0.01, ***P <* *0.001, ****P <* *0.0001.

Acute SCI often causes disruption of physiological homeostasis, which immediately leads to irreversible neuronal death and nerve degeneration [38]. Therefore, the neuronal differentiation of NSCs and their functionalization is vital for spinal cord regeneration and functional recovery [39]. In this study, biomarkers MAP2 and GFAP were used for identification of neuron-like and astrocyte-like differentiation of the NSCs cultured on different hydrogels, respectively, through immunofluorescence staining (Figure 4C). It was noted that the DSCM + NT3 hydrogel facilitated the NSCs neuronal differentiation, rather than the GFAP+ astrocyte-like cells, compared to the pristine DSCM hydrogels (Figure 4D and E). Surprisingly, the integrated curcumin was even more supportive for NSCs neuronal differentiation. The NSCs cultured on the DSCM + Cur hydrogel exhibited even more MAP2+ expression and much less GFAP+ expression than those identified on the DSCM + NT3 hydrogel. Such regulatory behaviors were consistent with a previously reported study, which demonstrated that curcumin can suppress the differentiation of NSCs into astrocytes while promoting their neuronal differentiation through decreasing acetylation of histone H3 and H4 [40]. Moreover, microscopical observation showed that the NSCs cultured on the DSCM + NT3 + Cur hydrogel resulted in superior number of MAP2+ cells and the least number of GFAP+ cells among all other hydrogels. The simultaneous release of both NT-3 and curcumin synergistically promoted the cultured NSCs differentiation into neurons (MAP2+) and inhibited their astrocyte-like (GFAP+) differentiation.

Previous studies have reported that NT-3 facilitates NSCs proliferation by activating TrkC receptor and promotes neurogenesis after SCI [41, 42]. On the other hand, curcumin has been also reported to positively contribute in NSCs neuronal differentiation, while suppresses their differentiation into astrocytes [40]. Apparently, our results indicated that the presence of both NT-3 and curcumin endowed the DSCM hydrogel with synergistic and beneficial effects in NSCs proliferation and differentiation.

### From anti-inflammatory to pro-regenerative: DSCM + NT3 + Cur hydrogel modulates phenotypes of macrophages and sequentially facilitates NSCs neuronal differentiation

**Figure 5.**
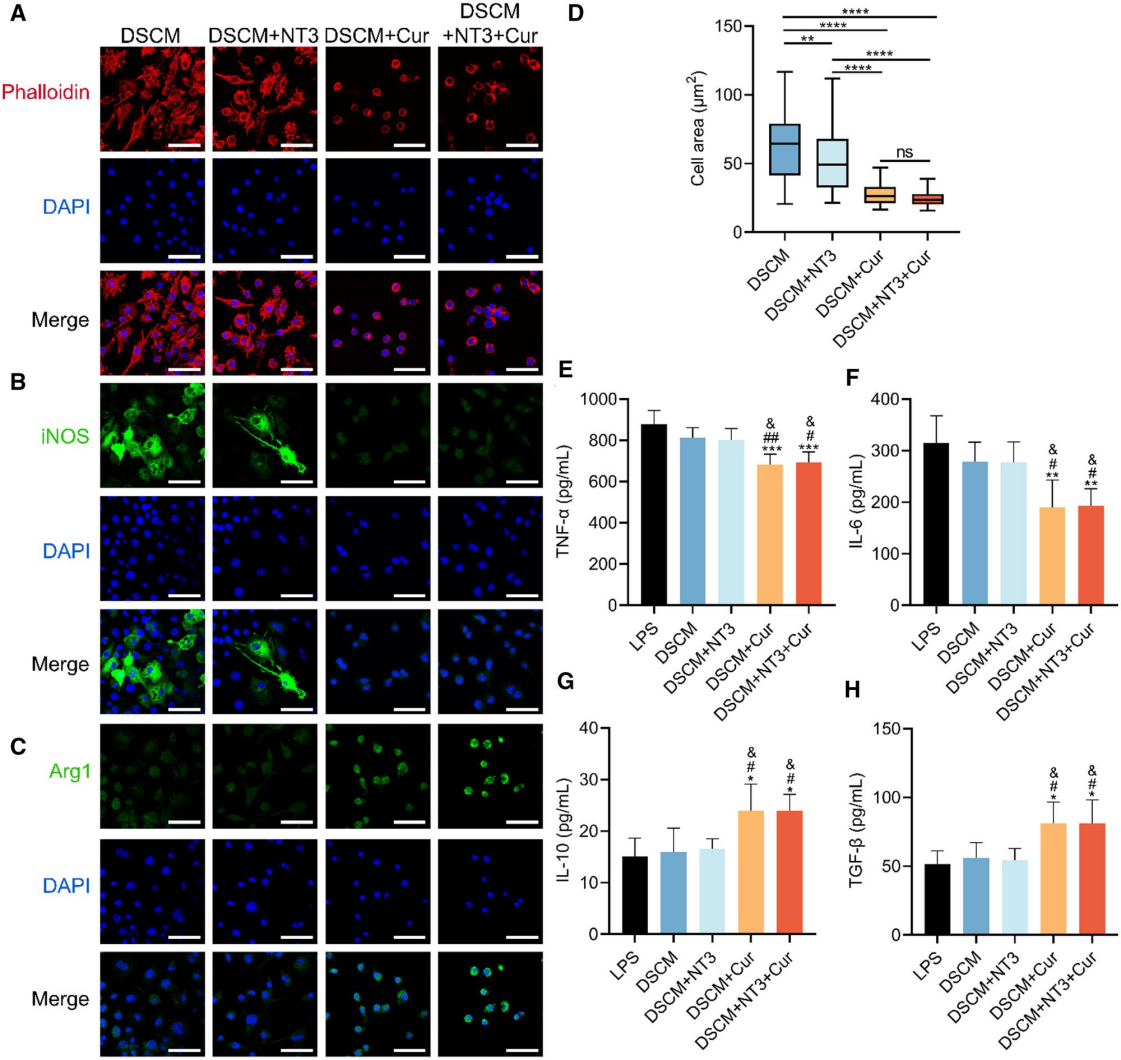
Phenotype transformation of macrophages when cultured on the DSCM, DSCM + NT3, DSCM + Cur and DSCM + NT3 + Cur hydrogels, respectively. (**A**) RAW 264.7 macrophages cultured on the hydrogels after LPS and IFN-γ stimulation, then immunostained by phalloidin and DAPI, scale bars = 20 μm. Immunofluorescence staining on the macrophages cultured on the hydrogels, after 24 h LPS and IFN-γ stimulation, using (**B**) iNOS and (**C**) Arg1 as the biomarkers for pro-inflammatory and anti-inflammatory cells, respectively, scale bars = 20 μm. (**D**) Cell spreading areas of the macrophages cultured on the hydrogels. Data were presented as the mean ± SD. The statistical significance was calculated by one-way ANOVA with a Tukey’s multiple comparison; ns not significant, **P <* *0.01, ****P <* *0.0001. ELISA analysis of (**E**) TNF-α, (**F**) IL-6, (**G**) IL-10 and (**H**) TGF-β after LPS and IFN-γ stimulation. The stimulated macrophages cultured on tissue culture plates was used as the LPS group. Data were presented as the mean ± SD. The statistical significance was calculated by one-way ANOVA with a Tukey’s multiple comparison; *n* = 5 for all groups; *P <* *0.05, **P <* *0.01 and ***P <* *0.001 represent comparison with the LPS group, ^#^*P *<* *0.05 and ^##^*P *<* *0.01 represent comparison with the DSCM group, &*P *<* *0.05 represents comparison with the DSCM + NT3 group.

After SCI, the subsequent inflammatory responses activated by infiltrated monocytes, macrophages and microglia often lead to secondary damage to the injured nerve tissues [43]. Particularly, the phenotypes of macrophages and their transformation play a crucial role in modulating the neuroimmune microenvironment for SCI repair [44]. To investigate the phenotype modulation in response to different DSCM hydrogels, macrophages were pre-stimulated by LPS and IFN-γ for 24 h before culturing on the DSCM, DSCM + NT3, DSCM + Cur and DSCM + NT3 + Cur hydrogels, respectively. After 3 days of culture, phalloidin was employed for observation of cytoskeleton and morphological changes of the macrophages. It was noted that the macrophages cultured on DSCM and DSCM + NT3 hydrogels exhibited larger amount of F-actin expression (labeled by phalloidin), implicating significantly increased cell area and isotropic cytoplasmic/pseudopod extension (Figure 5A). The macrophages cultured on the curcumin-containing hydrogels (DSCM + Cur and DSCM + NT3 + Cur), however, only showed round-shaped cells with limited F-actin expression. Statistically, the macrophages cultured on the DSCM + Cur and DSCM + NT3 + Cur hydrogels occupied much less area compared to those on the DSCM or DSCM + NT3 hydrogels (Figure 5D), again indicating the modulation of macrophages phenotypes through integration of curcumin.

Previous studies suggested that the distinct morphologies of macrophages are intimately correlated with their phenotypes. To further evaluate the influence of the hydrogels on immunoregulation of macrophages, biomarkers iNOS and Arg1 were used to characterize the phenotype modulation of the LPS and IFN-γ stimulated macrophages (Figure 5B and C). The pro-inflammatory iNOS+ expression was easily identified in the macrophages cultured on the curcumin-free hydrogels, especially on the DSCM alone hydrogel, the mean fluorescence density of iNOS+ expression was much greater than those on the other hydrogels (Supplementary Figure S4A). Conversely, little-to-no iNOS+ signal was observed on the hydrogels that integrated with curcumin. On the other side, most of the cells cultured on the DSCM + Cur and DSCM + NT3 + Cur hydrogels were labeled by Arg1, a classic biomarker for anti-inflammatory macrophages, whereas the intensity of Arg1+ fluorescence emission was very weak on both DSCM and DSCM + NT3 hydrogels (Supplementary Figure S4B).

To verify the effects of macrophage polarization on the hydrogels, the secretion levels of specific inflammatory-related cytokines were examined by ELISA, including the pro-inflammatory TNF-α/IL-6 and anti-inflammatory IL-10/TGF-β, which correspond to the M1 and M2 macrophage phenotypes, respectively (Figure 5E–H). The stimulated macrophages cultured on the hydrogel-free tissue culture plates were employed as the control group (denoted as ‘LPS’ in Figure 5E–H). It was noted that the macrophages cultured on both curcumin-containing hydrogels (DSCM + Cur and DSCM + NT3 + Cur) significantly decreased the amount of TNF-α and IL-6 secretion, while increased the expression of both IL-10 and TGF-β factors. These results confirmed that the curcumin integrated DSCM hydrogels exhibited prominent capability in modulating the cultured macrophages from pro-inflammatory to anti-inflammatory phenotype.

**Figure 6.**
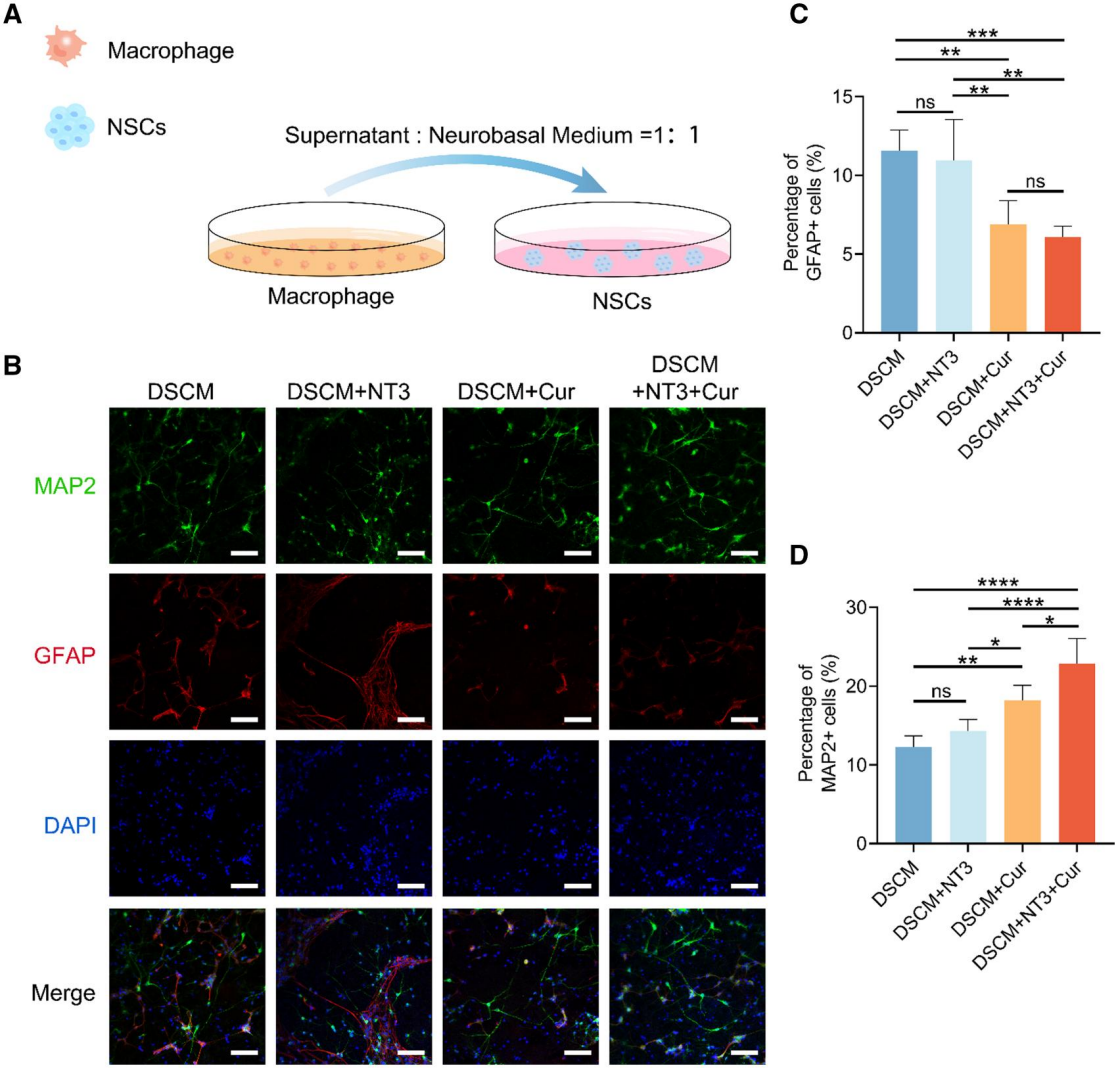
NSCs differentiation induced by macrophages-secretion containing culture media. (**A**) Schematic diagram of NSCs cultured in the mixed medium (supernatant from macrophage medium: neurobasal medium = 1: 1). (**B**) NSCs were cultured on the hydrogels using the mixed medium for 14 days, then immunostained with MAP2, GFAP and DAPI, scale bars = 100 μm. Percentage of (**C**) GFAP+ and (**D**) MAP2+ cells among all the cultured NSCs. Data were presented as the mean ± SD. The statistical significance was calculated by one-way ANOVA with a Tukey’s multiple comparison; *n* = 5 for all groups; ns represents not significant, *P <* *0.05, **P <* *0.01, ***P <* *0.001, ****P <* *0.0001.

It has been acknowledged that different macrophage phenotypes can further regulate NSCs differentiation through secretion of various cytokines [45, 46]. To examine the cell responses to the inflammatory environment resulted from the simulated macrophages on different hydrogels, the supernatant from each macrophage medium aforementioned was extracted and mixed with the Neurobasal medium at a ratio of 1:1 for culturing NSCs (Figure 6A). After 14 days of culture, it was noticed that the supernatants from macrophage culture media significantly regulated the cultured NSCs differentiation. Obviously, the anti-inflammatory macrophages on both curcumin-containing hydrogels resulted in more pro-regenerative microenvironment for NSCs differentiation, since more MAP2+ cells and much less GFAP+ cells were identified when NSCs were cultured in the DSCM + Cur and DSCM + NT3 + Cur groups, compared to the DSCM and DSCM + NT3 groups (Figure 6B–D). Moreover, besides the unipolar MAP2+ neurons, more multipolar neurons were observed when culturing in the mixed medium, compared to those identified in the neurobasal medium alone (Supplementary Figure S5). It has been well-acknowledged that axonal regrowth and extension are essential for functional restoration after SCI [47]. Meanwhile, multifunctional recovery also depends on the diversity of regenerating neurons. Compared to unipolar neurons, multipolar neurons are more difficult to survive, which usually take extra time for their consequential axonal regeneration [48]. Our finding preliminarily indicated that the secretion of stimulated macrophages cultured on the DSCM + NT3 + Cur hydrogel provided a more suitable microenvironment for the survival of multipolar neurons, which kept a relatively more diverse cell resources for functional nerve regeneration.

The DSCM + NT3 + Cur hydrogel led to a superior culture environment that not only benefited NSCs neuronal differentiation but also suppressed their differentiation into astrocytes, partly owing to the dual-released NT-3 and curcumin, which also attributed to the paracrine effects of the anti-inflammatory/pro-regenerative macrophages. Overall, these results suggested that DSCM + NT3 + Cur hydrogel remodeled the culture microenvironment for NSCs proliferation, transformation of macrophages from a pro-inflammatory to an anti-inflammatory phenotype, and eventually favored their neuronal differentiation and maturation, implicating its regenerative potential in SCI repair.

### *In situ* injected DSCM + NT3 + Cur hydrogel provides anti-inflammatory microenvironment toward functional recovery after contused SCI in rats

**Figure 7.**
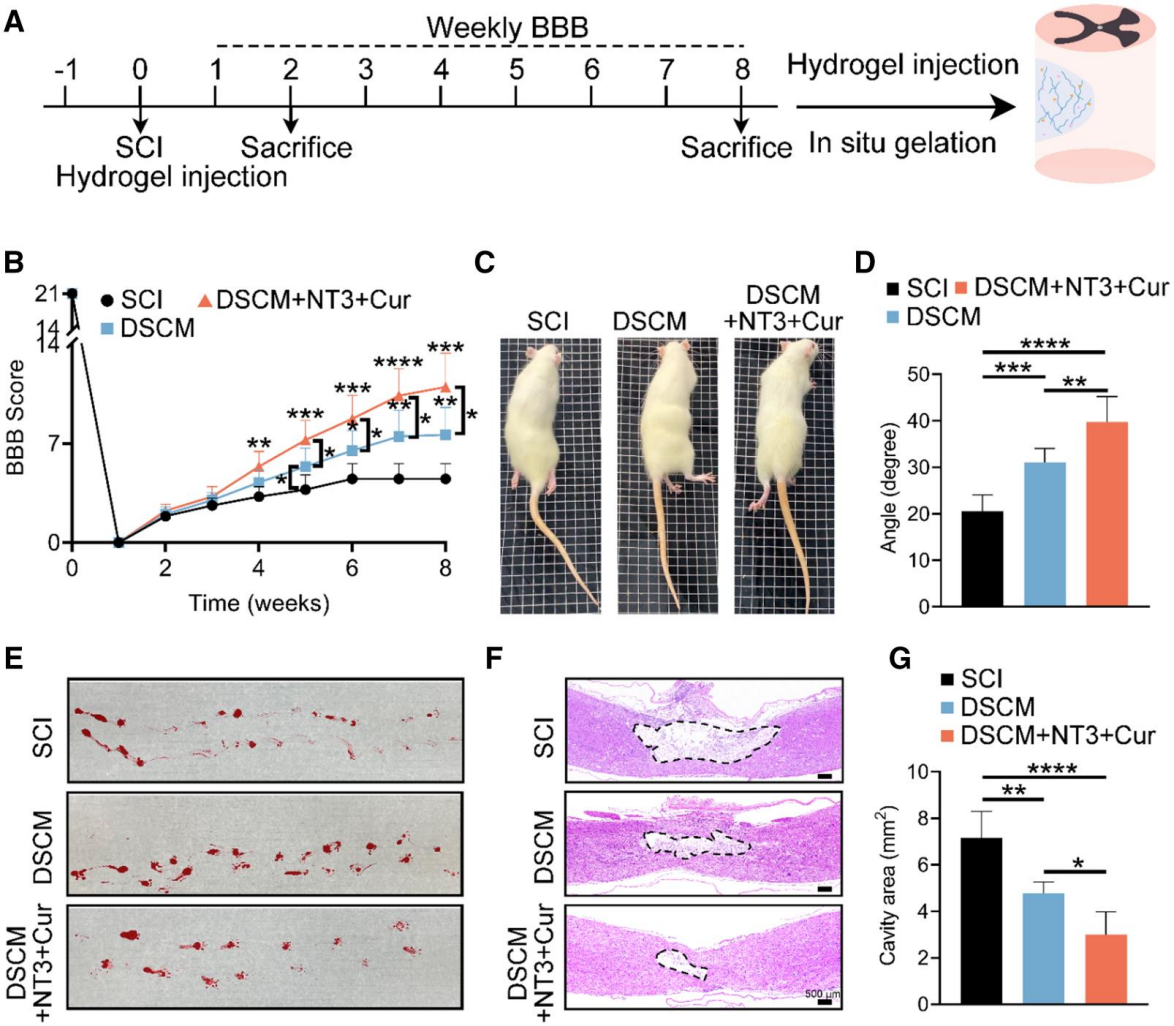
Functional recovery of contused SCI rats after *in situ* injected DSCM + NT3 + Cur hydrogel. (**A**) Summary of experimental design and schematic diagram of timeline. (**B**) BBB open-field walking scores of rats in SCI, DSCM and DSCM + NT3 + Cur groups. Data are presented as the mean ± SD. The statistical significance was calculated by two-way ANOVA with a Tukey’s multiple comparison; *n* = 6 for all groups; *P <* *0.05, **P <* *0.01, ***P <* *0.001. (**C**) Representative images of the rats climbing on inclined grids 8 weeks after SCI. (**D**) Angles of the inclined plates for rat climb-up. Data were presented as the mean ± SD. The statistical significance was calculated by one-way ANOVA with a Tukey’s multiple comparison; *n* = 6 for all groups; **P <* *0.01, ***P <* *0.001, ****P <* *0.0001. (**E**) Representative footprints of the rats 8 weeks post-injury. (**F**) Representative micrographs showing the H&E staining of the dissected spinal cords in each group 8 weeks after injury. (**G**) Quantitative analysis of the total area of cavity at the lesion sites. Data were presented as the mean ± SD. The statistical significance was calculated by one-way ANOVA with a Tukey’s multiple comparison; *n* = 3 for all groups; *P <* *0.05, **P <* *0.01, ****P <* *0.0001.

For *in vivo* assessments, the DSCM + NT3 + Cur hydrogel was injected *in situ* into the contused spinal cords in rats (DSCM + NT3 + Cur group), then compared with the untreated (SCI group) and the ones treated with DSCM hydrogel alone (DSCM group). The summary of the experimental design and schematic diagram are illustrated in Figure 7A. First, the Basso-Beattie and Bresnahan (BBB) open-field locomotor score was employed to evaluate the recovery of hind limb motor function weekly after SCI (Figure 7B). Within 1 week after contusion, all the rats displayed complete hind limb paralysis with BBB scores declined to 0. The BBB scores of the rats without hydrogel treatment reached a plateau at ∼6 weeks post-injury. Conversely, the rats in both DSCM and DSCM + NT3 + Cur groups exhibited persistent locomotion recovery throughout 8 weeks after SCI. Especially, the rats in the DSCM + NT3 + Cur group showed superior locomotor functional recovery among all three groups after 4 weeks. The inclined grid climbing tests showed that the rats of SCI group only climbed on their forelimbs with minor knee movements observed (Figure 7C and Supplementary Video S1). However, the rats of both DSCM and DSCM + NT3 + Cur groups can climb up the grids using their hind limbs cooperatively (Figure 7C, Supplementary Videos S2 and S3). The angles of the inclined plates used for both DSCM and DSCM + NT3 + Cur groups were statistically much larger than those of the SCI group (Figure 7D). Meanwhile, the rats of the DSCM + NT3 + Cur group showed clearest footprints during walking, implicating significant locomotion functional recovery 8 weeks after hydrogel injection (Figure 7E).

Eight weeks post-injury, the biosafety of both administrated hydrogels was first examined by H&E staining on the spleens, kidneys and livers of the treated animals. It was clearly evident that these vital organs exhibited similar histological morphologies as those in the health rats at the same age, indicating good biocompatibility and no toxicity of both DSCM and DSCM + NT3 + Cur hydrogels *in vivo* (Supplementary Figure S6). Meanwhile, H&E histological analysis showed that the hydrogel-injected spinal cords possessed significantly reduced volume of cavity compared to those of the untreated SCI group, especially, the smallest cavitation was observed in the DSCM + NT3 + Cur groups (Figure 7F and G). Furthermore, the extent of collagen scar formation was evaluated by Masson trichrome staining (Supplementary Figure S7). Although collagen deposition was evident in all groups after contused SCI, the *in situ* injection of DSCM + NT3 + Cur hydrogel resulted in the most attenuated collagen scar formation.

**Figure 8.**
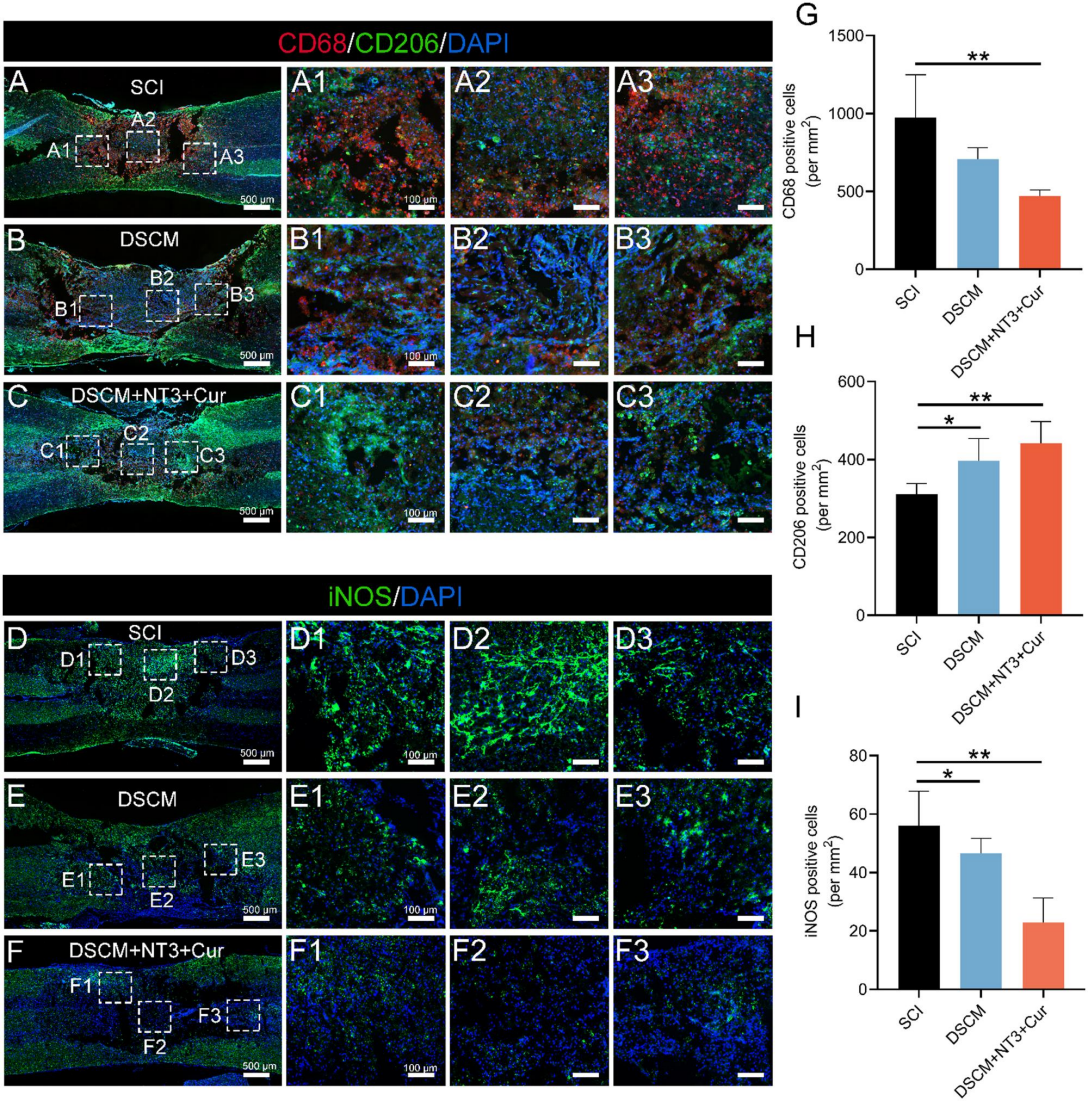
*In situ* injected DSCM + NT3 + Cur hydrogel suppressed pro-inflammatory responses after contused SCI. (**A**–**C**) Representative micrographs show the injured spinal cords in the (A) SCI, (B) DSCM and (C) DSCM + NT3 + Cur groups 2 weeks post-SCI, immunofluorescence co-stained by CD68, CD206 and DAPI, scale bars = 500 μm for the lower magnification images on the left-hand side. Micrographs (A1–3), (B1–3) and (C1–3) on the right-hand side show the zoom-in images of the corresponding boxed regions, scale bars = 100 μm. (**D**–**F**) Representative micrographs show the injured spinal cords in the (D) SCI, (E) DSCM and (F) DSCM + NT3 + Cur groups 2 weeks post-SCI, immunofluorescence stained by iNOS and DAPI, scale bars = 500 μm for the lower magnification images on the left-hand side. Micrographs (D1–3), (E1–3) and (F1–3) on the right-hand side show the zoom-in images of the corresponding boxed regions, scale bars = 100 μm. (**G**–**I**) The densities of (G) CD68+, (H) CD206+ and (I) iNOS+ cells at the lesion sites in SCI, DSCM and DSCM + NT3 + Cur groups, respectively. All data were presented as the mean ± SD. All analyses were implemented using one-way ANOVA with a Tukey’s multiple comparison; *n* = 5 for all groups; *P <* *0.05, **P <* *0.01.

It was widely acknowledged that SCI triggers innate inflammatory events with immediate infiltration of inflammatory cells into the lesion site, which further forms a harsh microenvironment and hinders nerve regeneration. Therefore, controlled regulation of inflammatory responses in the early stage has been a valid therapeutic approach [49], in which case, the phenotype modulation of microglia and macrophages becomes critical in treatments of SCI [50]. In this study, a general biomarker CD68 was used for identification of macrophages/microglia, co-stained with biomarker CD206 for their anti-inflammatory phenotypes to assess different subpopulation of the activated macrophage/microglia at the lesion sites of all groups 2 weeks after contused SCI (Figure 8A–C). It was noted that numerous CD68+ cells accumulated in the connective tissues with few cells co-stained with CD206 in the SCI group. In contrast, the number of CD206+ cells significantly increased in both hydrogel-treated groups (Figure 8G and H). Particularly, the injection of DSCM + NT3 + Cur suppressed the penetration of overall CD68+ cells, among which most of the macrophages/microglia were identified to be CD206+, indicating their anti-inflammatory phenotype. Further immunofluorescence staining was applied to examine the inflammatory response after contused SCI using biomarker iNOS, the results clearly showed that accumulated iNOS+ signal was observed at lesion sites of the SCI rats, while much fewer iNOS+ cells were identified in the DSCM + NT3 + Cur group (Figure 8D–F and I). The administrated DSCM hydrogel alone slightly decreased the total number of iNOS+ cells, but still exhibited insufficient anti-inflammatory effects compared to the DSCM + NT3 + Cur hydrogel. These results were consistent with the *in vitro* studies that the DSCM + NT3 + Cur hydrogel facilitated the transformation of macrophage/microglia phenotypes from pro-inflammatory to anti-inflammatory. Such positive modulation of inflammation was attributed to the introduction of DSCM hydrogel, as well as the release of curcumin *in situ*.

### DSCM + NT3 + Cur hydrogel recruits endogenous NSCs and suppresses astrocyte scar formation for facilitating nerve regeneration

**Figure 9.**
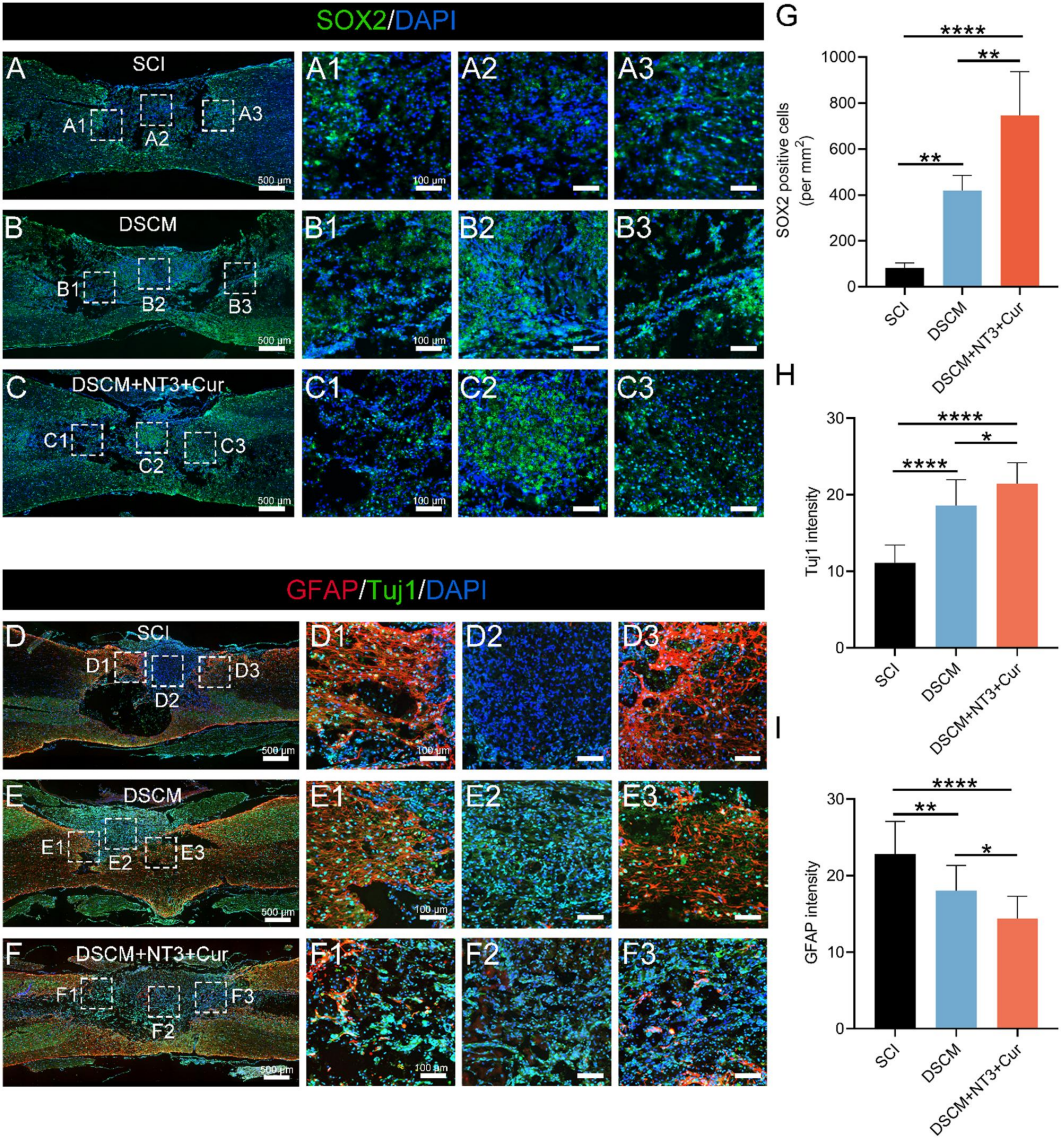
Recruitment of endogenous NSCs and nerve regeneration after contused SCI and *in situ* hydrogel treatments. Representative micrographs show the results of SOX2 and DAPI immunofluorescence staining at the lesion sites of (**A**) SCI, (**B**) DSCM and (**C**) DSCM + NT3 + Cur groups, respectively, 2 weeks post-injury. Representative micrographs show the results of co-immunostained GFAP, Tuj1 and DAPI at the lesion sites of (**D**) SCI, (**E**) DSCM and (**F**) DSCM + NT3 + Cur groups, respectively, 8 weeks post-injury. Scale bars = 500 μm for the lower magnification images on the left-hand side. Micrographs (1–3) on the right-hand side show the zoom-in images of the corresponding boxed regions, scale bars = 100 μm. (**G**) The density of SOX2+ cells at the lesion site of the spinal cords in SCI, DSCM and DSCM + NT3 + Cur groups, respectively. (**H** and **I**) Quantitative analysis of optical density for Tuj1 and GFAP. All data were presented as the mean ± SD. Analyses were implemented using one-way ANOVA with a Tukey’s multiple comparison; *n* = 5 for all groups; *P <* *0.05, **P <* *0.01, ****P <* *0.0001.

Recruiting endogenous NSCs to the lesion site is central to nerve regeneration regarding cell-free SCI repair strategies [45–47]. Our previous study demonstrated that DSCM hydrogel promoted endogenous NSCs migration and their functionalization after completely transected SCI, but the nerve functional recovery was limited [10]. As an important supplement to the DSCM hydrogel in this study, the neurotrophic factor NT-3 can facilitate migration of endogenous NSCs into the injury site and reconstruct the damaged neural network [20]. To validate this idea, immunofluorescence biomarker SOX2 was employed to label the endogenous NSCs around the lesion sites 2 weeks after contused SCI (Figure 9A–C). It was noted that large amount of SOX2+ cells were easily identified in the DSCM and DSCM + NT3 + Cur groups, while much fewer NSCs were observed at the lesion sites of the SCI group, especially at the epicenter of the contused regions. Statistically, the largest SOX2+ cell population was identified by the DSCM + NT3 + Cur group compared to the DSCM group, and much greater than that of the SCI group (Figure 9G), indicating that the NT-3 containing hydrogel significantly enhanced the recruitment of endogenous NSCs.

**Figure 10.**
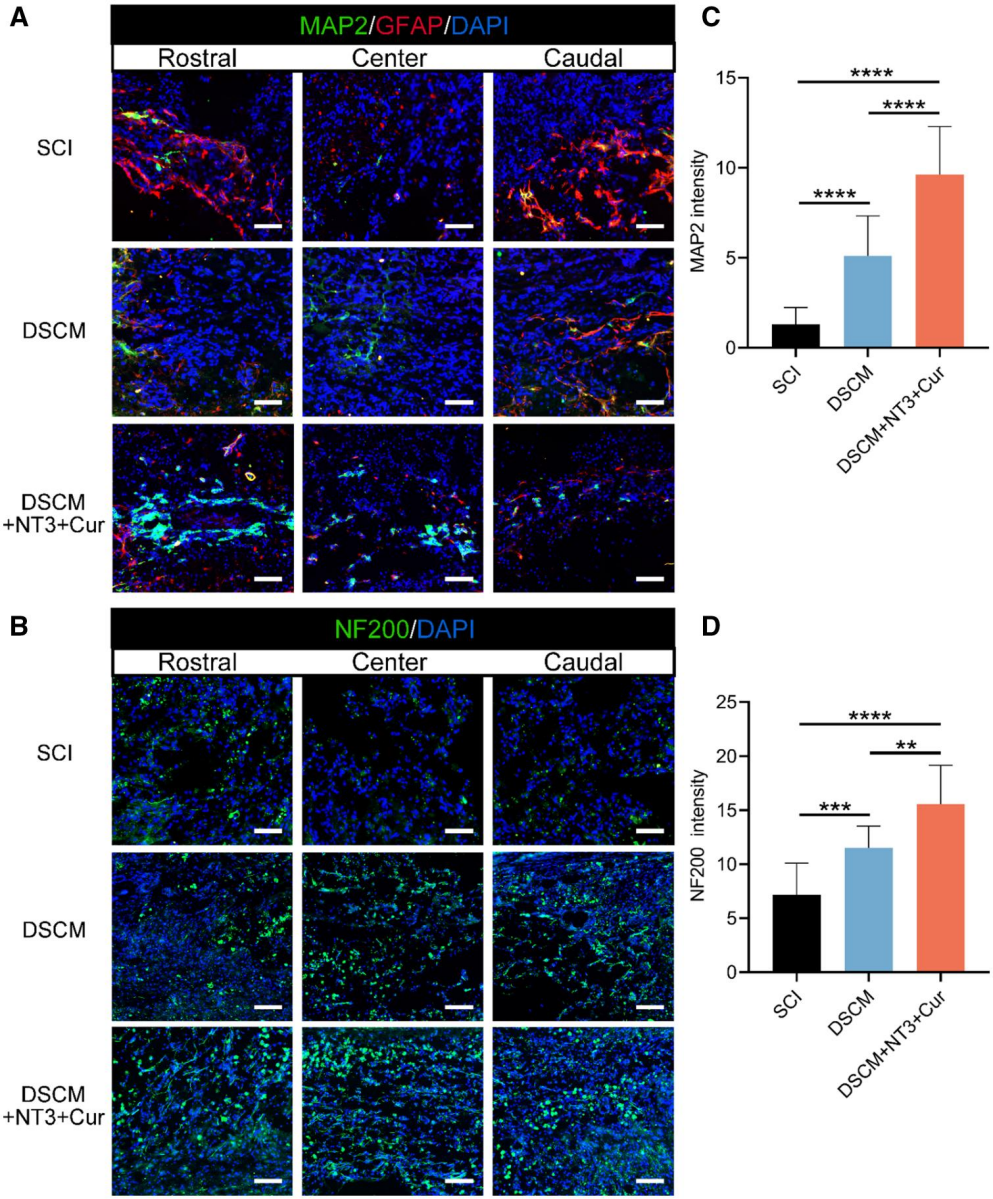
Evaluation of nerve regeneration 8 weeks after contused SCI and *in situ* hydrogel treatments. (**A**) Representative micrographs show the results of immunofluorescence staining using biomarkers MAP2, GFAP and DAPI at rostral, center and caudal sites, respectively. (**B**) Representative micrographs show the results of co-immunostained NF200 and DAPI at the injury sites, respectively. Scale bars = 100 μm. Quantitative analysis of optical densities of the (**C**) MAP2+ and (**D**) NF200+ immunofluorescence signals. All data were presented as the mean ± SD. Analyses were implemented using one-way ANOVA with a Tukey’s multiple comparison; *n* = 5 for all groups; **P <* *0.01, ***P <* *0.01, ****P <* *0.0001.

Following recruitment of NSCs, effective nerve regeneration requires replenishing neurons through manipulation of NSCs differentiation, as well as reducing glial scar deposition, which eventually contributes to neural circuit reconstruction. To identify the neuronal regeneration capacity after hydrogel treatments, the lesion sites of the spinal cords were co-immunostained by neuronal β-tubulin III (Tuj1) and GFAP to localize newborn neurons and astrocytes, respectively (Figure 9D–F). It was evident that significant amounts of Tuj1+ cells were accumulated at the injury sites of both DSCM + NT3 + Cur and DSCM hydrogel treated spinal cords, while little Tuj1 expression was identified in the SCI group (Figure 9H). Meanwhile, it was noticed that much fewer GFAP+ astrocytes were observed around or within the lesion sites of the DSCM + NT3 + Cur group. In contrast, the GFAP+ cells were much denser surrounding the injury sites of the other two groups (Figure 9I). Especially, GFAP+ glial scars were found heavily accumulated at both rostral and caudal ends of the injured area in the SCI groups, while sparse scar formation was observed in the DSCM + NT3 + Cur group (Supplementary Figure S8). The modulation of host scarring response between implant and host is key for nerve regeneration [51]. The highly alleviated formation of glial scars provided abundant space for endogenous NSCs infiltration. To further evaluate the neuronal regeneration capacity after *in situ* hydrogel administration, biomarkers MAP2 and NF200 were used to identify mature neurons and nerve fibers at the lesion sites through immunostaining (Figure 10A and B). The MAP2 expression in the injury site of DSCM + NT3 + Cur group was significantly superior to that of the SCI and DSCM group (Figure 10C), suggesting that the drug-loaded hydrogel highly promoted the maturation of recruited endogenous NSCs. Meanwhile, in contrast with the SCI and DSCM group, abundant NF200+ neurites were observed to distribute consecutively at the lesion sites of the DSCM + NT3 + Cur group (Figure 10B). Statistically, the fluorescence intensity of NF200+ signal in the DSCM + NT3 + Cur group was significantly higher than that in the SCI and DSCM groups (Figure 10D).

Collectively, it was evident that the sustained co-delivery of NT-3 and curcumin in the DSCM + NT3 + Cur hydrogel cooperatively modulated the lesion site into a pro-regenerative microenvironment, which eventually facilitated nerve regeneration and functional recovery after contused SCI. The DSCM hydrogel alone showed its great potential serving as a biocompatible drug-delivery cargo for *in situ* administration, as well as a bioactive microenvironment regulator after SCI. Although curcumin has shown assets in modulating inflammation, considering its limited water solubility, nanocarrier-assisted curcumin delivery systems can be rationally designed and developed prior to integration into DSCM hydrogel, including nanoparticles, microspheres, liposomes and exosomes, to further augment its therapeutic effects [52–54].

## Conclusions

To endow the *in situ* injectable hydrogel with multifunctional bioactivities, NT-3 and curcumin were simultaneously integrated into the tissue-specific DSCM hydrogel. The neurotrophic factor and anti-inflammatory drug were intimately encapsulated in the hydrogel, and both additives followed a sustained release manner. The controlled release of NT-3 served actively in endogenous NSCs recruitment, proliferation and functionalization. In the meantime, the integrated curcumin contributed mainly to the transformation of macrophages from pro-inflammatory to anti-inflammatory phenotype, which further facilitated NSCs neuronal differentiation, and suppressed their differentiation into astrocytes and glial scar formation thereafter. The co-delivery of NT-3 and curcumin worked cooperatively in reconstructing a pro-regenerative microenvironment at the lesion sites after contused SCI, which effectively promoted nerve regeneration and locomotor functional recovery after hydrogel injection. We believe that the current study not only presents a promising strategy for multifunctional treatment of SCI, but it also provides a springboard for developing advanced *in situ* drug-delivery systems using dECM hydrogels.

## Supplementary materials

Supplementary data are available at *Regenerative Biomaterials* online.

## Supplementary Material

rbae039_Supplementary_Data
