# Supplementary material for: Sustained delivery of NT-3 and curcumin augments microenvironment modulation effects of decellularized spinal cord matrix hydrogel for spinal cord injury repair
Source: Regen Biomater. 2024 Apr 10;11:rbae039. doi: 10.1093/rb/rbae039 (PMC11090998; doi:10.1093/rb/rbae039)
Supplement: rbae039_Supplementary_Data [file rbae039_supplementary_data.docx]

Supplementary Information

**Sustained delivery of NT-3 and curcumin augments microenvironment modulation effects of decellularized spinal cord matrix hydrogel for spinal cord injury repair**

Jiaxin Chen^a^, Xing Cheng^b^, Zhengran Yu^b^, Rongli Deng^a^, Rui Cui^a^, Jing Zhou^a^, Houqing Long^b^, Yong Hu^c^, Daping Quan^a,^*, Ying Bai^a,^*

^a^ Guangdong Engineering Technology Research Centre for Functional Biomaterials, Key Laboratory for Polymeric Composite & Functional Materials of Ministry of Education, School of Materials Science and Engineering, Sun Yat-sen University, Guangzhou 510006, China

^b^ Department of Spine Surgery, The First Affiliated Hospital of Sun Yat-sen University, Guangzhou 510080, China

^c^ Department of Orthopedics and Traumatology, Li Ka Shing Faculty of Medicine, The University of Hong Kong, Pokfulam, Hong Kong, China

*Correspondence address. Tel: +86-20-84114030; Fax: +86-20-84112245.

E-mail: cesqdp@mail.sysu.edu.cn; baiy28@mail.sysu.edu.cn.

Table S1. Abbreviations

| Abbreviations | Full name |
| --- | --- |
| SCI | Spinal cord injury |
| NT-3 | Neurotrophin-3 |
| Cur | Curcumin |
| DSCM | Decellularized spinal cord matrix hydrogel |
| DSCM+NT3 | NT-3 integrated decellularized spinal cord matrix hydrogel |
| DSCM+Cur | Cur integrated decellularized spinal cord matrix hydrogel |
| DSCM+NT3+Cur | Decellularized spinal cord matrix hydrogel containing both NT-3 and Cur |
| ECM | Extracellular matrix |
| dECM | Decellularized extracellular matrix |
| G’ | Storage modulus |
| G’’ | Loss modulus |
| SEM | Scanning electron microscopy |
| CCK-8 | Cell counting kit-8 |
| ELISA | Enzyme-linked immunosorbent assay |
| EdU | 5-ethynyl-2’-deoxyuridine |
| LPS | Lipopolysaccharide |
| IFN-γ | Interferon gamma |
| PFA | Paraformaldehyde |
| H&E | Hematoxylin and eosin |
| BBB | Basso-Beattie-Bresnahan |
| OCT | Optimal cutting temperature |
| NSCs | Neural stem cells |
| NSPC | Neural stem/progenitor cell |
| M1 macrophage | Pro-inflammatory macrophage |
| M2 macrophage | Anti-inflammatory macrophage |
| SEM | Scanning electron microscopy |
| SOX2 | Recombinant sex determining region Y box protein 2 |
| Tuj1 | Tubulin β-III |
| MAP2 | Microtubule-associated protein 2 |
| GFAP | Glial fibrillary acidic protein |
| iNOS | Inducible nitric oxide synthase |
| Arg1 | Arginase-1 |
| TNF-α | Tumor necrosis factor-α |
| IL-6 | Interleukin-6 |
| IL-10 | Interleukin-10 |
| TGF-β | Transforming growth factor-β |
| NF200 | Neurofilament-200 |

Table S2. Primary and secondary antibodies for immunofluorescence staining

| Antibodies | Species | Dilution | Company, product No. |
| --- | --- | --- | --- |
| Anti-Nestin (Nestin) | Mouse | 1:500 | Abcam, UK, ab254048 |
| Anti-microtubule-associated protein-2 (MAP2) | Rabbit | 1:500 | Sigma, USA, AB5622 |
| Anti-glial fibrillary acidic protein (GFAP) | Chicken | 1:500 | Abcam, UK, ab134436 |
| Anti-Sox2 (Sox2) | Rabbit | 1:500 | Abcam, UK, ab93689 |
| Anti-β-tubulin III (Tuj1) | Mouse | 1:500 | Sigma, USA, T8660 |
| Anti-CD68 (CD68) | Rabbit | 1:200 | Abcam, UK, ab283654 |
| Anti-CD206 (CD206) | Mouse | 1:500 | Abcam, UK, ab49999 |
| Anti-iNOS (iNOS) | Rabbit | 1:500 | Abcam, UK, ab178945 |
| Anti-Arg1 (Arg1) | Rabbit | 1:500 | Thermofisher, USA, PA5-85267 |
| Rhodamine Phalloidin |  | 1:800 | Solarbio, China, CA1610 |
| DAPI |  | 1:2000 | Santa Cruz, USA, sc-74421 |
| Alexa Fluor 488-conjugated  anti-mouse secondary antibody | Donkey | 1:800 | Thermofisher, USA, A32766 |
| Alexa Fluor 594-conjugated  anti-mouse secondary antibody | Donkey | 1:800 | Thermofisher, USA, A-21203 |
| Alexa Fluor 488-conjugated  anti-rabbit secondary antibody | Donkey | 1:800 | Thermofisher, USA, A-21206 |
| Alexa Fluor 594-conjugated  anti-rabbit secondary antibody | Donkey | 1:800 | Thermofisher, USA, A32754 |
| Alexa Fluor 594-conjugated  anti-chicken secondary antibody | Donkey | 1:800 | Thermofisher, USA, A78951 |


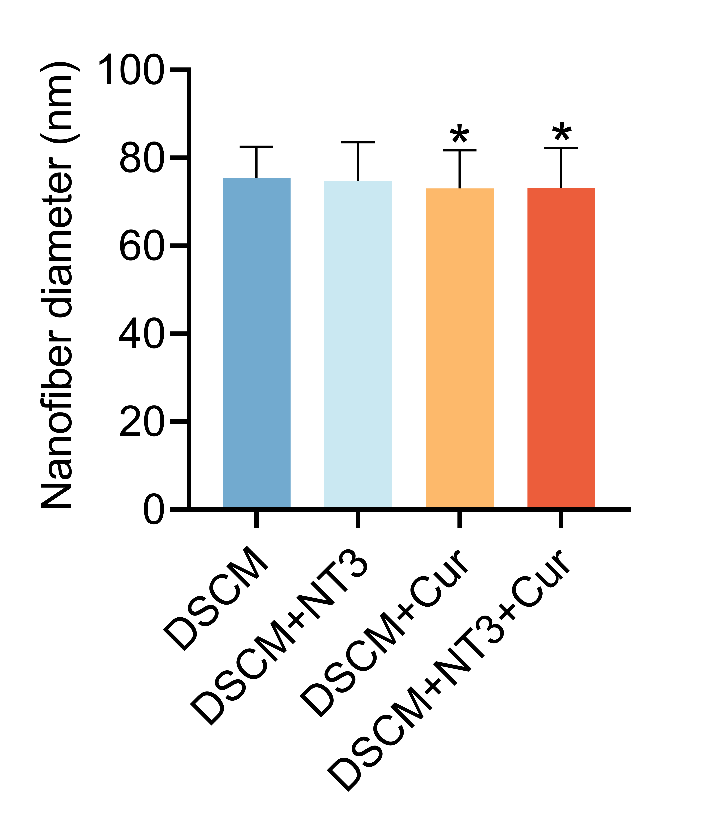


**Fig. S1.** The Diameter of the nanofibrous structure in DSCM, DSCM+NT3, DSCM+Cur, and DSCM+NT3+Cur hydrogels, characterized by SEM. **p* < 0.05, compared to DSCM hydrogel.


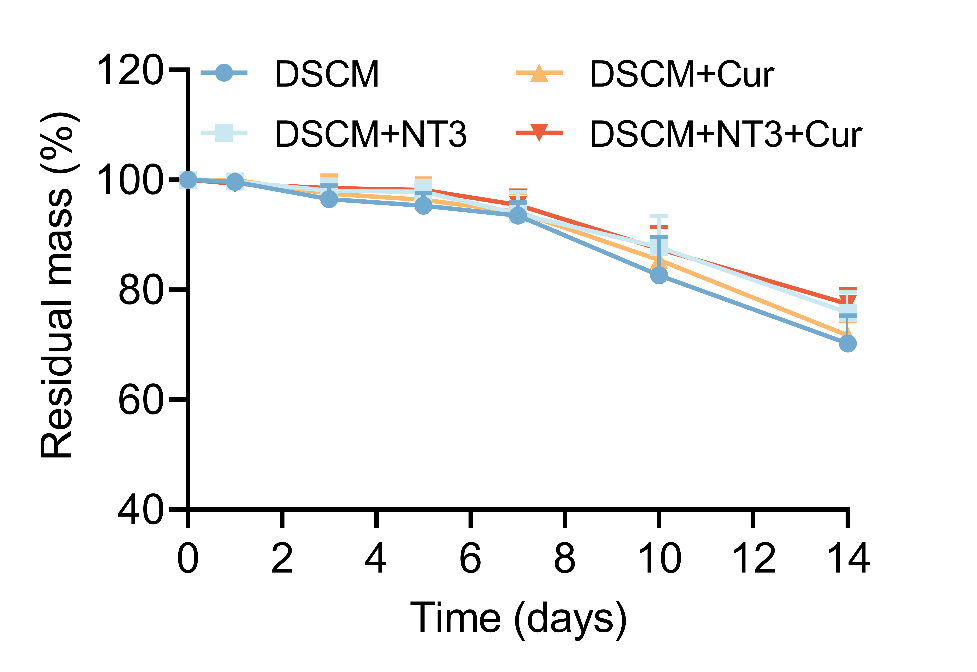


**Fig. S2.** Degradation behaviors of the DSCM, DSCM+NT3, DSCM+Cur, and DSCM+NT3+Cur hydrogels when immersed in PBS solution at 37 °C for two weeks, respectively. Presented as the residual mass of each hydrogel specimen with respect to immersion time.


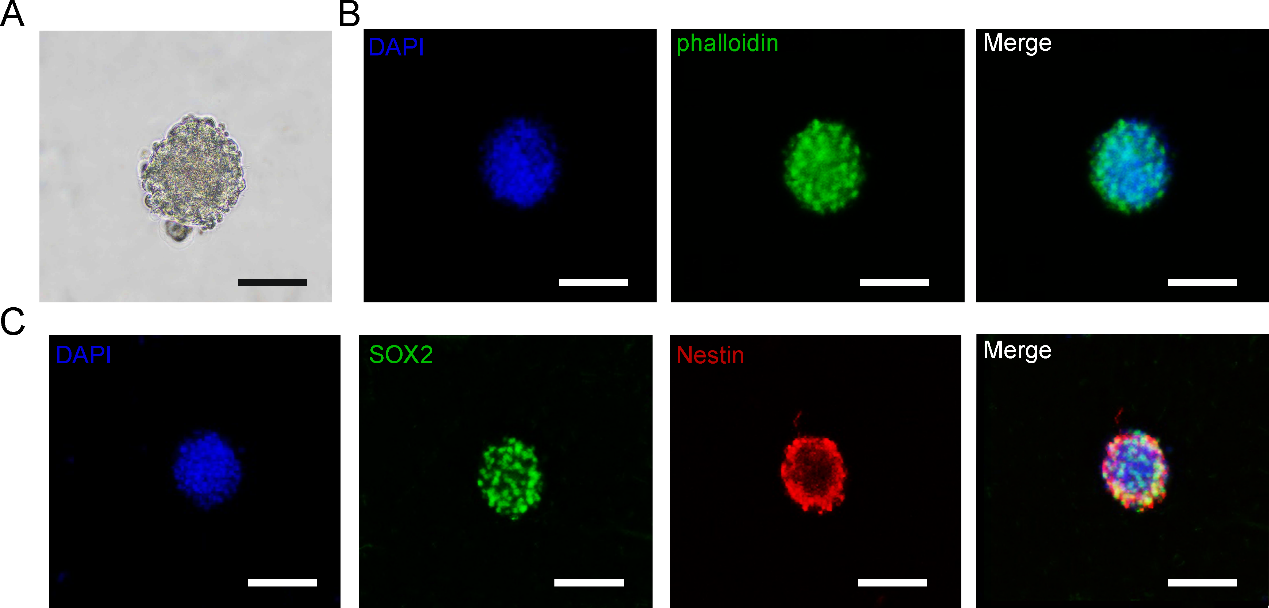


**Fig. S3.** (A) Optical micrograph of a representative primary NSC spheroid. (B) Representative fluorescence micrographs of the NSC spheroid show the nuclei (DAPI, blue) and cytoskeleton (phalloidin, green). (C) Representative Immunofluorescence staining of the NSC spheroid using biomarkers SOX2 (green), Nestin (red), and DAPI (blue). Scale bars = 100 μm.


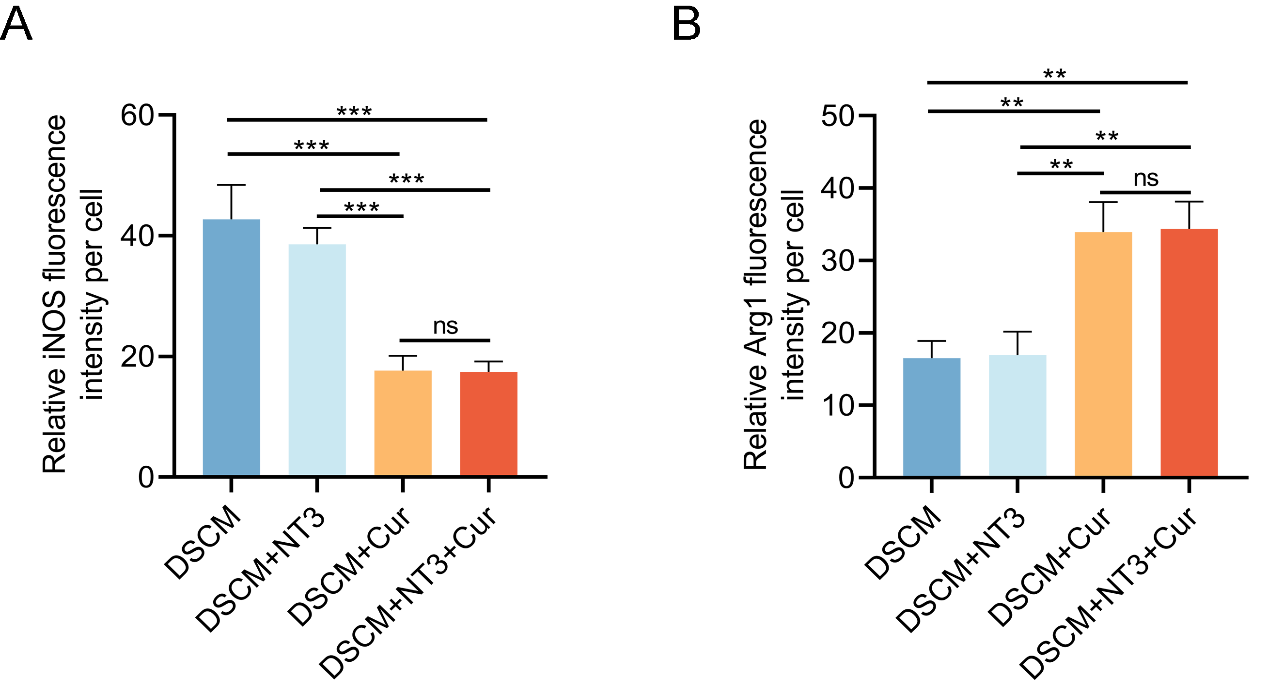


**Fig. S4.** Quantitative analysis of the fluorescence intensity of (A) iNOS and (B) Arg1, respectively. All data are presented as the mean ± SD. Analyses were implemented using one-way ANOVA with a Tukey’s multiple comparison; n = 3 for all groups; ns not significant, ***p* < 0.01, ****p* < 0.001.


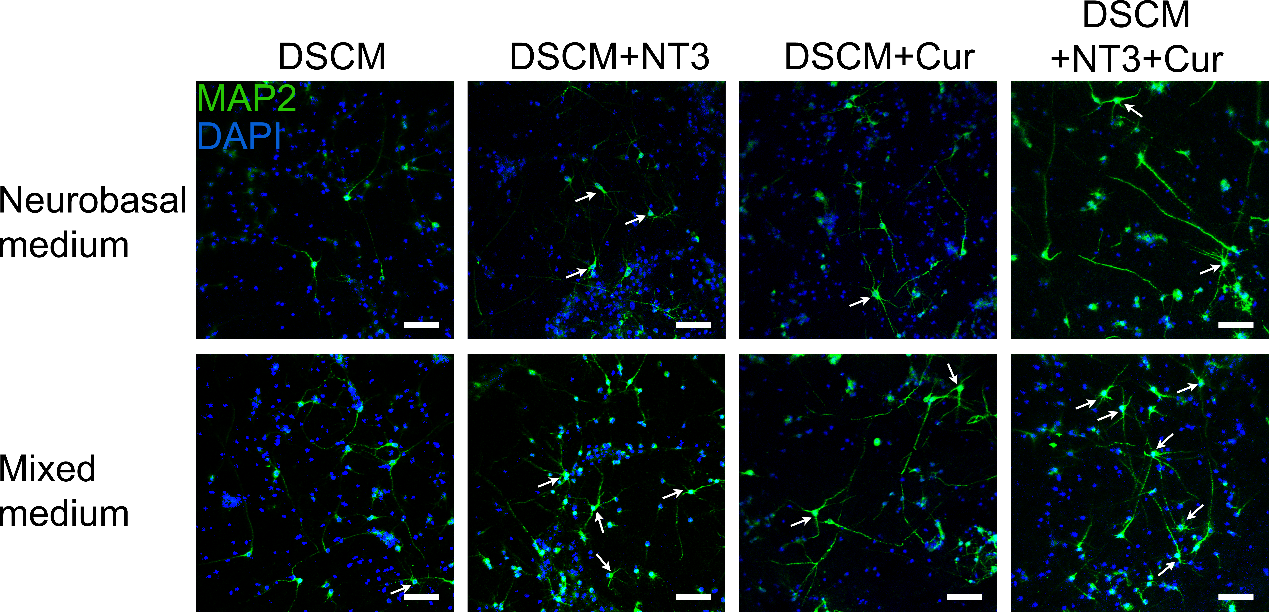


**Fig. S5.** The NSCs were cultured on the DSCM, DSCM+NT3, DSCM+Cur, and DSCM+NT3+Cur hydrogels using neurobasal medium alone and mixed medium (supernatant from macrophage medium: neurobasal medium = 1: 1) for 14 days, respectively, then subjected to immunofluorescence staining using MAP2 (green) and DAPI (blue). Scale bars = 100 μm. The white arrows point out the multipolar MAP2+ neurons.


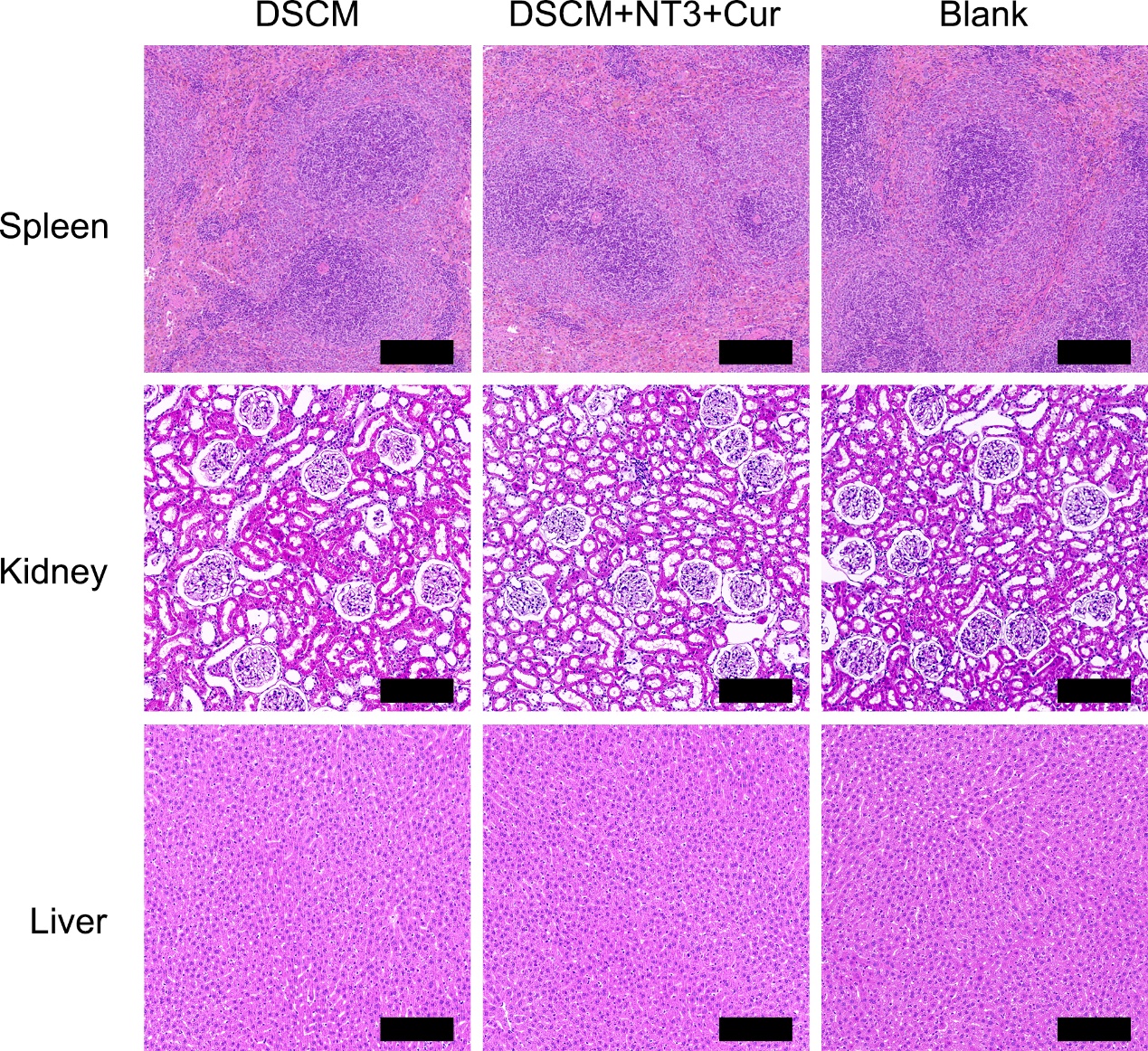


**Fig. S6.** Representative histological micrographs after H&E staining on the spleens, kidneys, and livers of both DSCM and DSCM+NT3+Cur groups eight weeks post-implantation. Scale bars = 200 μm.


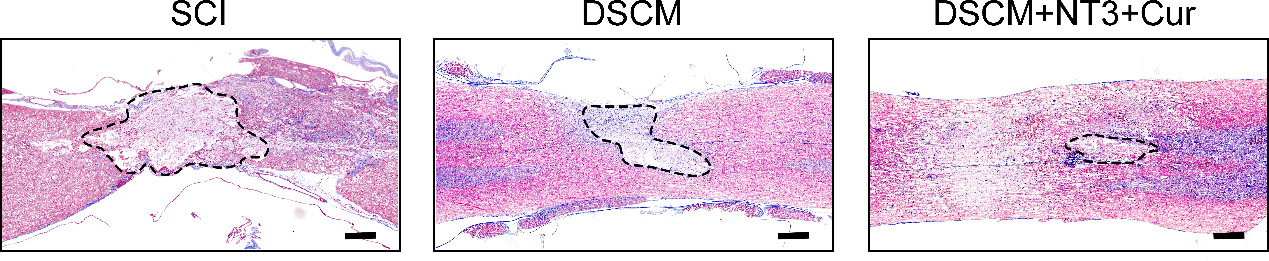


**Fig. S7.** Collagen scar formation at the lesion sites, characterized by Masson trichrome staining eight weeks after contused SCI. Scale bars = 500 μm.


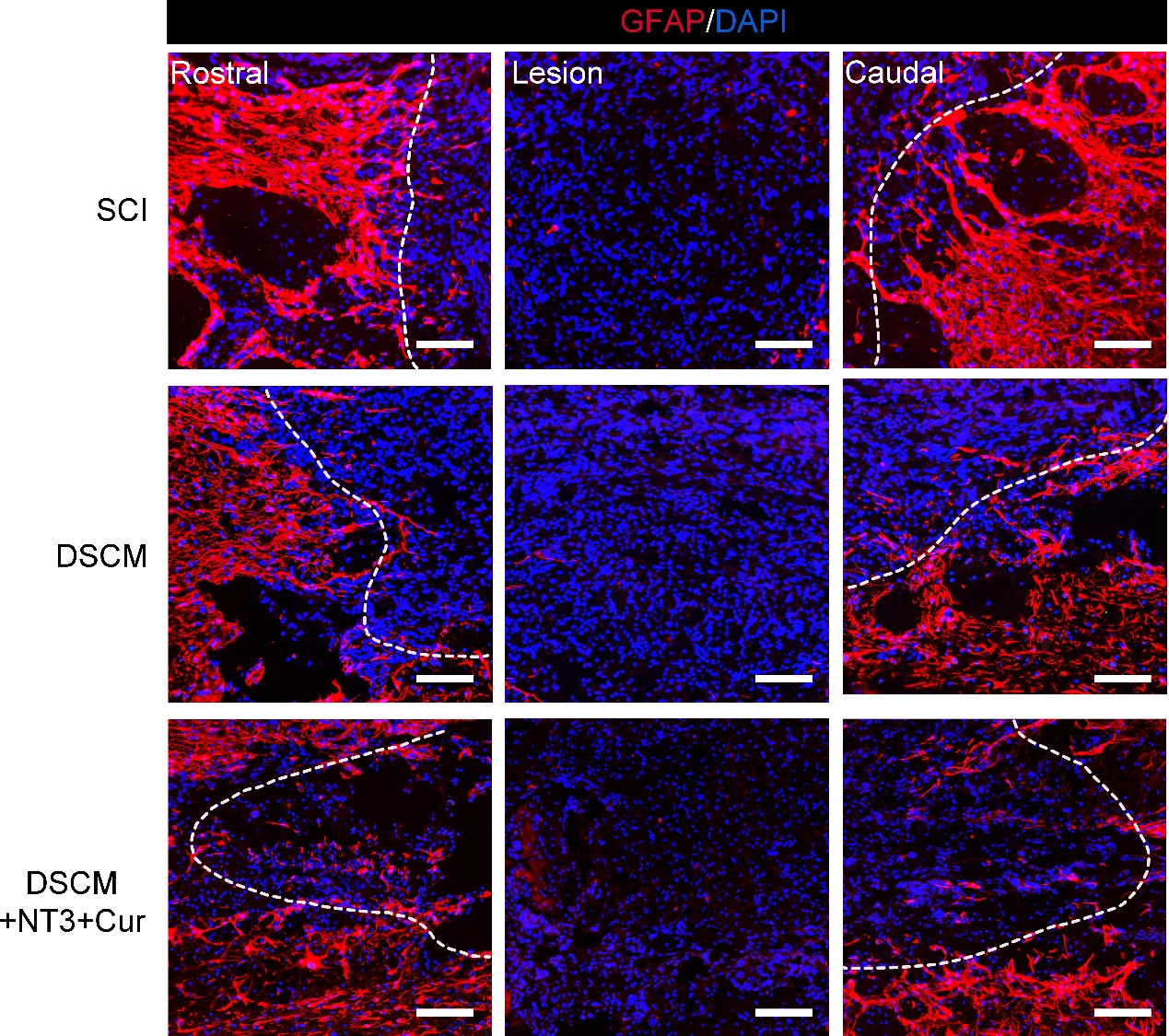


**Fig. S8.** Representative fluorescence micrographs showing the distribution of GFAP+ (red) glial scars at the rostral, lesion, and caudal regions in the injured spinal cords of SCI, DSCM, and DSCM+NT3+Cur groups eight weeks post-injury, scale bars = 100 μm.
